# Investigation of the Formation, Characterization, and Oxidative Catalytic Valorization of Humins

**DOI:** 10.3390/ma16072864

**Published:** 2023-04-04

**Authors:** André Wassenberg, Tobias Esser, Maximilian J. Poller, Jakob Albert

**Affiliations:** Institute for Technical and Macromolecular Chemistry, University of Hamburg, 20146 Hamburg, Germany

**Keywords:** sustainable chemistry, green catalysis, waste valorization, humins, polyoxometalates, biomass, synthesis and characterization

## Abstract

The industrial use of biomass, e.g., for the production of platform chemicals such as levulinic acid, became increasingly important in recent years. However, the efficiency of these processes was reduced by the formation of insoluble solid waste products called humins. Herein, the formation of humins from various carbohydrates was investigated under different process conditions, in order to obtain information about the structure and the formation mechanism. During this process, new potential structural fragments of humins were identified. Subsequently, the produced humins were oxidatively converted to low-molecular-weight carboxylic acids with the use of polyoxometalate catalysts. The experiments showed that the use of sugars in acetic acid and ethanol only lead to the formation of a small amount of humins, which were also structurally most suitable for conversion to carboxylic acids. The main products of the oxidative valorisation of these humins were acetic acid, formic acid, and CO_2_, respectively, and our results indicate that certain functional groups were converted preferentially. These findings will help to improve processes for the valorisation of biomass by enabling an overall more efficient use of thermo-sensitive feedstock such as carbohydrates.

## 1. Introduction

Increasing global energy consumption, growing waste generation, and collective environmental awareness in our society are driving the development of sustainable processes. In addition, the current dependence of energy and chemical production on fossil raw materials and the political powers that control them lead to an increasing importance of renewable raw materials such as biomass. These materials provide the possibility of an easy access to a variety of sustainable platform chemicals and secondary energy sources through innovative processes [1]. However, to date, not many technologies exist that can convert biomass into higher value chemicals. One reason is the formation of unwanted by-products by thermal-induced reactions that make such processes inefficient and expensive. An example for such a process is the synthesis of levulinic acid from sugars (Biofine-process). In the latter, insoluble black solid polymers are formed, which are called humins [2,3,4]. They are formed during the acid-catalyzed conversion of carbohydrates below a temperature of 250 °C [5,6,7,8,9]. Humins are particularly problematic for industrial processes, as they clog up both reactor and catalyst, and signify a waste of resources into an unusable side product [10,11,12,13,14,15].

For these reasons, researchers investigated strategies to prevent the formation of humins during the conversion of biomass. Particularly, the use of different solvents [16,17,18,19] or special acidic catalysts [20,21,22] were the focus of such research efforts. While these methods were successful in reducing the formation of humins [16], it remains impossible to avoid them entirely. Therefore, the valorization of humins was investigated as an alternative strategy. Different approaches were investigated so far: the use of humins for the production of synthesis gas [23,24,25], as a precursor for porous materials [26], as a biofuel additive, or energetic valorization by using them directly as a fuel [3]. A promising approach is the acid-catalyzed depolymerization of humins using zeolites [27,28] or polyoxometalates [4,29], as catalysts to produce humic oils as biofuel additives or low-molecular carboxylic acids. This more targeted conversion of humins into value added products showed a lot of potential for their future valorization.

**Figure 1:**
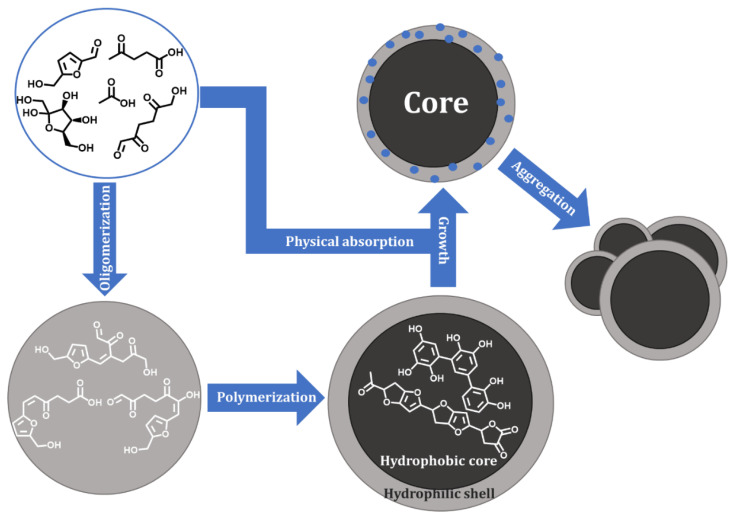
Process of humin formation from hexoses according to Siwei et al. [9].

In order to further develop methods for the avoidance of humin formation, it is essential to understand the mechanism behind, and to elucidate, the molecular structure of humins. Both were studied by various research groups over the past decade. The first concerted effort for their structural elucidation was made by several research groups at the beginning of the last decade [6,8]. Lund et al. first postulated a mechanism of aldol condensation between the acid-catalyzed sugar conversion intermediates 5-hydroxymethylfurfural (5-HMF) and 2,5-dioxo-6-hydroxyhexanal (DHH) [6]. This mechanism was based on prior research made by Horvat et al., who first described the reaction intermediate DHH being involved in the synthesis during the acidic conversion of hexoses [2]. Later, van Zandvoort et al. [8] expanded on these results and made a first structural proposal for more complex humins. This was also the first time it was suggested that humins are not only formed by aldol condensations, but also through other reaction types such as etherification, since substances such as levulinic acid and, additionally, added trihydroxybenzene was also incorporated into the humin structure. This was eventually confirmed by the subsequent research of multiple research groups such as Tsilomelekis et al. [13] and Shen et al. [30]. Numerous other insights were made concerning the formation of humins, resulting in the reaction mechanism for hexoses to humins by Siwei et al. in Figure 1 [9].

First, hexoses are converted to 5-HMF and intermediates such as DHH by Brönsted-catalyzed hydrolysis. Subsequently, these intermediates react through etherification and esterification to form water-soluble oligomers, which, in turn, are connected through further reactions such as etherification, esterification, acetalization, and aldol condensations to form insoluble, hydrophobic humin cores [30,31,32]. These cores also possess a hydrophilic shell which is instrumental for the further growth of humins, expanding the hydrophobic core. During this process, catalysts and solvent molecules are also incorporated into the humin structure through polymerization with oligomers or through absorption [11,13,33,34,35]. The resulting particles will then agglomerate step by step into a foam-like humin coke [9]. Although this research identified structural motifs of humins, details of their structure remain unknown due to their overall very complex composition. Furthermore, the influence on the formation conditions on the humin structure have not yet been thoroughly understood. We have now taken up the task to fill this gap by synthesizing humins from different sugars in various solvent/acid combinations and, subsequently, characterizing them. In contrast to previous investigations, our focus was not just the avoidance of humin formation but also the best possible valorization of the humins that are unavoidably formed. With respect to humin valorization, we took inspiration from the OxFA process, which oxidatively converts even complex biomass to formic acid using a polyoxometalate catalyst (H_8_PV_5_Mo_7_O_40_) and molecular oxygen [36,37]. This work was pioneered by Maerten et al. in 2017, who used several different polyoxometalates to convert humins to formic acid and acetic acid [29]. Similarly, we used H_8_PV_5_Mo_7_O_40_ to oxidatively convert humins that were synthesized using different sugars, solvents, and acids.

The goal of our research was to analyze the influence of the reactants on humin formation and their resulting structure to an unprecedented extent. For this purpose, we synthesized a large variety of humins through different combinations of acids and solvents. Additionally, we compared the oxidative valorization of these different humins using polyoxometalate catalysts, to identify which structural features are preferentially converted On the one hand, we aimed to identify ideal reagents for biomass conversion to avoid excessive humin formation. On the other hand, we aimed to gain the greatest value from unavoidable humin formation.

## 2. Materials and Methods

### 2.1. General Procedure for Humin Formation Experiments

For humin synthesis, all chemicals were purchased commercially and used without further purification. Acetic acid (glacial) and ethanol were obtained from VWR chemicals. Sulfuric acid (95–97%) and dimethyl sulfoxide were obtained from Grüssing GmbH. D(+)-xylose, D(+)-glucose, D(−)-fructose (all for biochemistry), and p-toluenesulfonic acid monohydrate were all obtained from Merck Millipore. Sucrose 99% was obtained from Alfa Aesar.

All experiments were conducted in a tenfold screening plant equipped with ten 20 mL Hastelloy C276 autoclaves and PTFE gaskets. All other peripheral plant components, such as valves, were made of stainless steel. This setup was able to reach reaction temperatures of up to 200 °C, and with magnetic stirring of up to 1000 rpm.

The humins formation experiments were based on the procedures of van Zandvoort et al. [6]. Several stock solutions containing either pure water or a water/organic solvent mixture in a 1:1 ratio and the chosen acidic catalyst were prepared and set to a pH of 2. Then 5 mmol of the chosen sugars were weighed into 10 mL glass liners. An amount of 5 mL of the chosen stock solution was then injected into the glass liner and the glass liner was inserted into the reactors. This acid/solvent/sugar mixture provided a molar H^+^/sugar ratio of 1:100. The reactors were then heated to 180 °C at a pressure of approx. 45 bar (synthetic air) and the reaction was allowed to run for 6.5 h. After the reaction, the humins were filtered and the liquid phases sent for analysis. The humins were then rinsed with water, dried, ground in a mortar, and cleaned with water for 24 h by soxhlet extraction. The humins were then dried for 24 h at 80 °C.

### 2.2. General Procedure for Catalytic Oxidation of Humins

For selective catalytic oxidation experiments of humins, the polyoxometalate catalyst H_8_PV_5_Mo_7_O_40_ (HPA-5) was synthesized according to previous studies [38,39]. The molar ratio of the catalyst components was analyzed by ICP-OES. For the used catalyst, a stoichiometry of 1.2 P/5.0 V/7.0 Mo was determined.

In a typical experiment, each autoclave was loaded with 75 mg humin as substrate in a 10 mL glass liner. A stock solution containing about 28 mmol L^−1^ vanadium in the form of a polyoxometalate catalyst was prepared. In case of H_8_PV_5_Mo_7_O_40_ catalyst, 1000 mg or 0.56 mmol of polyoxometalate, corresponding to 2.8 mmol of vanadium, was dissolved in 100 mL water. Each liner was filled with 7.5 mL of this stock solution, resulting in a carbon to vanadium ratio of about ca. 19 mol_Carbon_ mol^−1^_V_. The glass liners were then inserted into the reactors. After purging the autoclaves with 10 bar oxygen, ensuring pure oxygen atmosphere, the reactors were pressurized to a pre-pressure of about 26 bar. Subsequently, a reaction temperature of 90 °C and a stirring speed of 300 rpm for a better heat transfer were set. As a result of the temperature increase, the pressure rose to the desired reaction pressure of 30 bar and the stirrer speed was set to 1000 rpm.

After the reaction, each phase (gas, liquid, solid) was analyzed separately. For this purpose, gas samples were collected from the reactor before depressurization, and then, the liquid and solid phase were separated by filtration.

### 2.3. Analytical Methods

The amount of gaseous CO_2_ and CO was measured using a Varian GC 450 on a 2 m × 0.53 mm (ID) Shin-Carbon-column using a TCD detector.

For the quantitative analysis of the aqueous phase (determination of soluble reaction products such as formic acid, acetic acid, and succinic acid), a SHIMADZU HPLC system outfitted with an Aminex HPX-87H 300 mm 7.8 mm BIORAD Column and a refractive index detector from SHIMADZU (Kyoto, Japan) was employed. A 5 mmol L^−1^ concentration of aqueous sulfuric acid solution was utilized as the eluent. Samples were analyzed at flow rates of 0.5 mL min^−1^, 49 bar of pressure, and 45 °C.

A QATR^TM^-S single-reflection ATR was used to measure AT-FT IR spectra in attenuated total reflection (ATR) measurement mode (with a diamond prism). The spectra were plotted in origin and smoothed with the Savitzky–Golay method.

The following analyses were carried out by the respective service groups in the Department of Chemistry at Hamburg University:

- The nuclear magnetic resonance (NMR) spectra were measured using a Bruker AVANCEII 600 MHz spectrometer. Samples were prepared by mixing 0.4 mL of the filtered reaction solutions with 0.2 mL of D_2_O. Spectra of the reaction solutions can be found in the Supporting Information.
- Elemental analyses were carried out using the Model EA-3000 analyzer of Fa. EuroVector (Milano, Italy).
- SEM micrographs were recorded using a LEO Gemini 15505 from Zeiss (Oberkochen, Germany).
- MALDI-TOF measurements were taken with a Bruker ultrafleXtreme (Billerica, MA, USA) in positive reflector mode. A 2 mg/mL dispersion of the humic acid in water and a 20 mg/mL dihydroxybenzoic acid/TA30 (ACN/H_2_O 30/70 + 0.1%TFA) solution were prepared for the measurements. A total of 0.7 μL of both solutions were then mixed and left to crystallize.

## 3. Results and Discussion

### 3.1. Influence of Sugar, Acid, and Solvent on Humin Formation, Composition, and Structure

In order to study the formation of humins, four different sugars were chosen as starting materials: fructose, glucose, xylose, and sucrose. Fructose and glucose were chosen as hexoses, allowing a comparison between aldoses and ketoses. Xylose was chosen as a pentose, because pentoses were reported to undergo a different humin formation mechanism, in which furfural is mainly involved [12]. Sucrose was used as an example for a disaccharide. These sugars were dissolved in various solvent mixtures: pure water, which is the most environmentally friendly solvent and inexpensive, as well as 1:1 mixtures of water with ethanol and with dimethylsulfoxide (DMSO). Both ethanol and DMSO are alternative polar organic solvents, with ethanol as an example of a protic and DMSO as an example of an aprotic solvent. The formation of humins is usually impeded by non-aqueous solvents [9,18,40]; therefore, we expected a molar 1:1 mixture of water and the organic solvents to exhibit lower humin formation than pure H_2_O. The sugars and solvents were combined with three different acid catalysts: sulfuric acid (pK_a_ of −3) was chosen as a strong inorganic acid, which is also commonly used in industrial acid catalysis [3]. Para-toluene sulfonic acid (p-TSA) (pK_a_ of −2.8) was used as a strong organic acid, which is also known to facilitate the solubilization of biomass [41]. Additionally, acetic acid (AA) (pK_a_ of 4.75) was chosen as a typical weak acid, which is also of great interest as a green acid catalyst [42]. Table 1 provides an overview of the sugar/solvent/acid combinations, and the designations for the resulting humins, which were used throughout this study.

The reaction conditions (1 mol/L sugar, pH = 2, 180 °C, 6 h reaction time) were chosen based on previous investigations by van Zandvoort et al. [8]. These conditions were shown to produce high yield of humins without the humins taking on the form of a foamy coke, which occurs when using higher temperatures or concentrations. In order to prevent the solvent from evaporating, a pressure of 45 bar of a 1:4 O_2_/N_2_ mixture was applied (for details, see Section 2). The initially homogeneous solutions were not stirred during the reaction time, because humin formation would have caused the stirrers to seize in the small reaction vessels.

In order to determine sugar conversion (Table 2), all reaction solutions were investigated via magnetic resonance spectroscopy (^1^H- and ^13^C-NMR) as well as high performance liquid chromatography (HPLC). All relevant spectra can be found in Figures S1–S16 in the Supporting Information. Most of the NMR-spectra showed no sugar signals, which corresponds to a conversion of 100%. In cases where sugar was still present, the residual amount of sugar was quantified by HPLC to determine the conversion. Fructose and xylose achieved full conversion in all reactions, while glucose and sucrose showed lower conversions in water. This confirmed van Zandvoort’s results [8]. The different conversions of glucose and fructose can be explained by the fact that fructose directly forms HMF and DHH, since it possesses a more stable ring structure, while glucose must first isomerize to fructose before reacting further to HMF and DHH [5,7,43,44]. Accordingly, sucrose also has a lower conversion, being a dimer of glucose and fructose, which must first be hydrolyzed into the monomeric sugars during the conversion process, with subsequent isomerization of glucose to fructose and conversion of the latter. This interpretation is supported by the NMR spectra (Figures S1 and S2 in the Supporting Information), as the sugar signals of the glucose and sucrose reaction solutions are identical. As the other sugars are completely converted, an effect of the acid can only be observed for the conversion of glucose in the following trend: stronger acids lead to higher conversion.

The NMR spectra additionally allowed the identification of certain intermediates (Figures S1 and S2 in the Supporting Information). Large amounts of formic acid, acetic acid, and levulinic acid were found in the aqueous reaction solutions of hexoses and sucrose. In addition, small amounts of 5-HMF were found in the NMR spectra of the acetic acid-catalyzed reaction solutions. This was a remarkable observation because weaker acids usually result in lower HMF yields [45,46], but here, HMF was only found in the reaction with the weakest acid. We assumed that during the reaction catalyzed by strong acids, all 5-HMF was polymerized to humins. In the EtOH/H_2_O mixtures, the same intermediates were found, next to additional compounds resulting from reactions with the solvent, such as diethyl ether, acetic acid, and ethyl acetate (Figures S7 and S8 in the Supporting Information). Using a DMSO/H_2_O mixture, formic acid was the only intermediate observed. However, additional signals here indicate decomposition products of DMSO, such as dimethyl sulfide and dimethyl disulfide (Figures S15 and S16 in the Supporting Information). This also indicates that DMSO is not stable under the applied reaction conditions.

**Figure 2:**
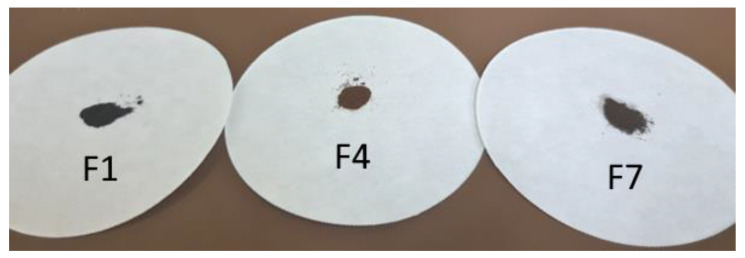
Humins obtained from fructose in different solvents using sulfuric acid as strong Brönsted acid.

The humins formed in these experiments were washed with water by Soxhlet extraction to remove any adsorbed soluble intermediates and were, subsequently, dried at elevated temperatures, ultimately resulting in powdery solids with a brown or black color (Figure 2). The syntheses in the purely aqueous solutions and the DMSO containing solutions resulted in black humins, while the syntheses in the solutions containing ethanol resulted in brown humins.

**Figure 3:**
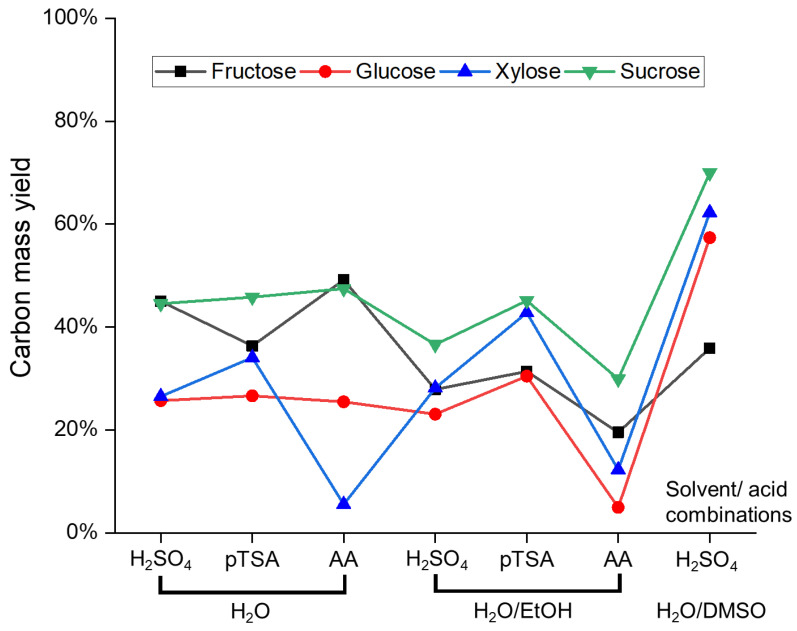
Overview of yields in humin formation experiments (determined with Equation (S1)).

Although the achieved conversion rates were high, the yield of humins (Figure 3) remained comparatively low, indicating that a large amount of the initial carbon remained in soluble intermediates. Generally, experiments using fructose (F1–F7) and sucrose (S1–S7) provide higher yields than glucose (G1–G7) and xylose (X1–X7), respectively. For glucose, this corresponds to the lower conversion of the sugar (Table 2). The reason for the lower humin yield resulting from xylose might be its shorter chain length, which impedes the formation of stable cyclic structures, and results in more soluble oligomeric intermediates.

This trend was not observed in reactions performed in DMSO (F7, G7, X7, and S7); however, in these cases, a substantial amount of solvent (and decomposition products) was incorporated into the humin structure (vide infra), thereby skewing the yield which was calculated relative to the initial amount of carbon in the sugar. In pure water, similar yields (approx. 45%) were obtained from sucrose (S1–S3) for all acids used. In the ethanol/water mixture, p-TSA showed a similar yield (S5), but H_2_SO_4_ (S4) and acetic acid (S6) formed less humin. The latter effect was also observed for glucose and fructose, whereas with xylose, all acids resulted in a higher yield in ethanol/water. In EtOH/H_2_O, the yield of all sugars, with the exception of xylose, decreased significantly when acetic acid was used. This might partially be caused by esterification of acetic acid with ethanol, making the catalyst less effective. The humin yields of the reactions using the weaker acid p-TSA were generally higher than those using the stronger sulfuric acid. However, the yields of F6, G6, X6, and S6 were all lower than those of using p-TSA and sulfuric acid. Although weaker acids should provide greater humin yields, this was the case, even though the usage of weaker acids should provide greater humin yields [45,46]. The lower yields were also consistent with the literature, as organic solvents were known to reduce the yield of humins [9,18,40]. It was, therefore, assumed that more soluble oligomers were formed by reactions of the intermediates of the sugar conversion with the resulting acetic acid and ethanol. These oligomers were then absorbed into the humin but were lost during purification and were, therefore, not considered when determining the yield. This was particularly evident with G6, where no solid was recovered in two subsequent replication attempts. The increased formation of soluble oligomers would also explain why, despite the lower humin yield, complete conversion of all sugars in the organic solvents was observed (Table 2). However, the xylose humins were excluded from this general trend, as their yields in organic solvents were greater than in pure water. Interestingly, this directly contradicted a study by Köchermann et al. which obtained lower yields from a similar mixture of xylose in ethanol and sulfuric acid than from a water/sulfuric acid mixture [47].

In summary, for the goal of avoiding humin formation, the use of acetic acid in EtOH/H_2_O using glucose as a substrate (G6), and the use of acetic acid in H_2_O for xylose (X3), provided the best results with a humin yield of only around 5%. Aside from DMSO, which was the worst solvent of the tested selection, fructose and sucrose in neat water resulted in the highest humin formation regardless of the acid used.

With regard to the valorization of humins, their elemental composition and structural characteristics are of great importance. Therefore, we analyzed a selection of the obtained humins by elemental analysis (CHSO), electron microscopy (SEM), infrared spectroscopy (IR), and matrix-assisted laser desorption time-of-flight mass-spectrometry (MALDI-TOF MS).

**Figure 4:**
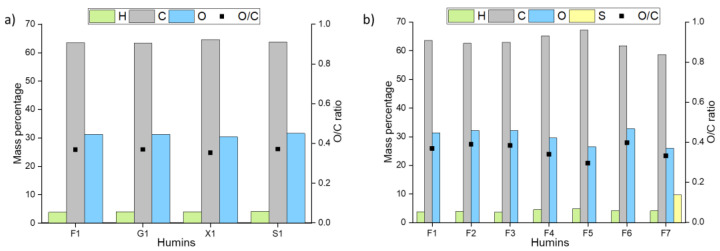
Elemental analysis of synthesized humins; (**a**) humins synthesized with sulfuric acid in water (**b**) humins synthesized from fructose using different acids and solvent mixtures (for elemental composition of used sugars, see Table S2).

The elemental composition of the synthesized humins from the different sugars was almost identical (Figure 4); only the xylose humin deviated slightly, as it possessed slightly lower H/C and O/C ratio. This confirmed the research carried out by van Zandvoort et al. in 2013. [8]. Their investigations showed that the elementary composition of the humins obtained from fructose and glucose hardly differed from one another. It is no surprise that sucrose as a dimer of fructose and glucose produced the same result.

Very interestingly, various acidic catalysts and solvents had a significant effect on the elemental composition of the resulting humins. Most notable was the presence of a significant sulfur content of about 10% for F7, which was likely due to DMSO and its decomposition products being incorporated into the humin structure. Together with the slightly lower carbon content and a hydrogen content which is similar to the other humins, this indicates the formation of thioethers/-esters in the humin structure. For humins synthesized in neat water, the oxygen content increases with decreasing strength of the acidic catalyst. This indicates that during humin formation weaker acids are more likely to catalyze esterification or etherification than aldol condensation. However, in the ethanol/water mixture, the oxygen content initially decreased sharply between sulfuric acid and p-TSA, and then, again rose up to the highest level at the weakest acid. The oxygen content of F6 here corresponded closely to that of F3. This similarity could be due to the large amount of acetic acid in both reaction solutions. Presumably, insoluble oligomers were increasingly formed here from the acetic acid and the sugars or intermediates, in which mostly ether or ester bonds were present, leading to an increased oxygen ratio. In contrast, F5 stands out due to possessing the highest hydrogen and carbon content of all fructose humins. This would indicate a high number of alkyl bonds in the humin, which could possibly indicate that, under these reaction conditions, primarily aldol condensations could be responsible for the humin formation. To a lower extent, this applies to F4 as well.

**Figure 5:**
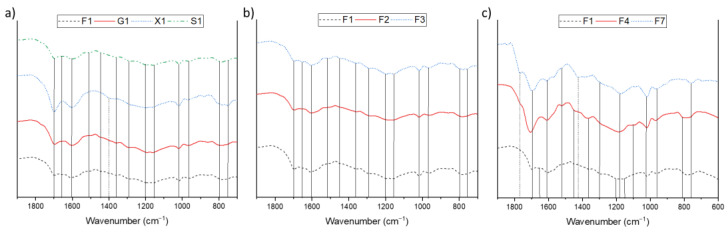
IR-spectra of different humins; (**a**) humins of different sugars catalyzed by sulfuric acid in neat water (**b**) fructose humins using different acids in water (**c**) fructose humins catalyzed with sulfuric acid in various solvents.

In order to further elucidate structural differences of the humins, the materials were further investigated by infrared spectroscopy (Figure 5).

In the IR spectra of all humins, the typical signals of substituted furans (C=C vibrations at 1600 cm^−1^ and 1510 cm^−1^, C-H out of plane vibrations at 750 cm^−1^ and 795 cm^−1^) were observed, which are common for humins from sugars [6,8,9,13,25,48]. Additionally, ether, carbonyl, and aliphatic chains were identified (Table 3). Sulfur containing structural features, e.g., thiols, were identified for the humins formed in DMSO (Figure 5c and Figures S17–S20 in the Supporting Information).

As expected, the spectra of humins formed from the hexoses and from sucrose were all very similar (Figure 5a). The spectrum of the xylose-based humin, on the other hand, differed significantly from the hexoses in three signals: the 1700 cm^−1^ band was much more pronounced here than with the other sugars, the 1670 cm^−1^ carbonyl vibrational bands were absent, and the signal of the framework vibrations was also shifted by approx. 35 cm^−1^. The carbonyl vibration was usually caused by the inclusion of 5-HMF into the humin structure, its absence was to be expected since humins from pentoses form via furfural and not 5-HMF [6,9]. The large difference concerning the 1700 cm^−1^ band could indicate that fewer aldol condensations were involved in the formation of xylose humins than in the formation of humins of other sugars. This different bonding pattern in the humin presumably also caused the shift in the framework vibration.

Comparing the humins formed by different acids, no major differences were observed in the IR spectra (Figure 5b). Only minor variations, such as the 1700 cm^−1^ band of F2 being slightly smaller, were identified. This suggests that the greater proportion of oxygen in this humin can be attributed to increased presence of ether bonds in the structure. The 1670 cm^−1^ band of F2 was also less pronounced. This matches the prior observation of the acetic acid catalyzed humins taking up all of the formed 5-HMF.

The biggest differences were found when comparing the humins derived from different solvents (Figure 5c). For the humin formed in DMSO (F7), two additional bands were found at 1775 cm^−1^ and 1420 cm^−1^. The former was presumably a signal caused by thioesters, while the latter was a signal from a C=S stretching vibration. These were caused through the incorporation of decomposition products of DMSO that were taken into the humin structure, as previously discussed. In addition, the signal of the furan out of bound oscillation at 795 cm^−1^ was no longer present with F7. This indicates that the specific furan structures were not present in this specific humin in contrast to the other humins. This probably also resulted from reactions with the sulfur-containing compounds. The 1700 cm^−1^ band was significantly weaker for F1 than for F5 and F7, corresponding to an increased number of esters and carbonyls in the latter materials. This indicates a larger number of esters and ethers being formed in ethanolic solution. This also explains the more intense peak at 1090 cm^−1^ compared with the other humins, since this signal corresponded to the C-O-C ether vibration of ethyl ethers. In contrast, the 1670 cm^−1^ carbonyl vibrational band was absent in both humins derived from partially organic solvents. This could either be due to the fact that less 5-HMF was involved in the humin formation or that the signal was overshadowed by the much larger 1700 cm^−1^ bands.

**Figure 6:**
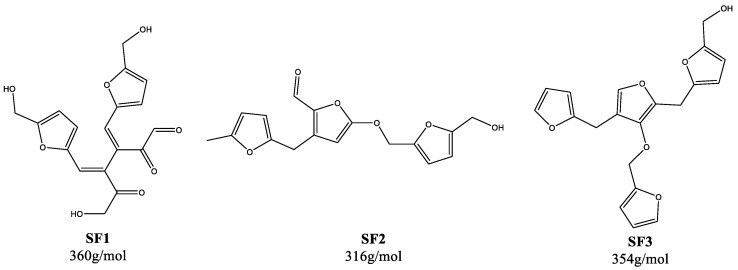
Postulated humin fragments based on mass spectra.

For additional structural investigations, selected humins (F1-3, G1, X1, and S1) were analyzed by MALDI-TOF MS (Figures S21–S32 in the Supporting Information). The goal was to identify common structural fragments by their m/z ratio; therefore, the main focus was the identification of similarities in the spectra. Although we were not able to detect the structural fragments postulated by Hoang et al. or Chen et al. [25,49], three fragments could be identified that repeatedly appeared in the humins (Figure 6).

Fragment SF1 with a mass of 360 g/mol was the product of the aldol condensation of DHH with two HMF molecules. A similar fragment, consisting of DHH and HMF was previously reported by Chen et al. [49]. This interpretation was further confirmed by the fact that the relevant peak did not appear in the mass spectrum of the xylose humin, since HMF and DHH did not form the basis for humin formation here. The other two fragments with 316 g/mol and 354 g/mol, respectively, were more difficult to assign. Despite the different degradation mechanisms of hexoses and pentoses, they both appeared in humins based on hexoses as well as xylose. The proposed structures SF2 and SF3 were based on that fact and on previous analytical results.

**Figure 7:**
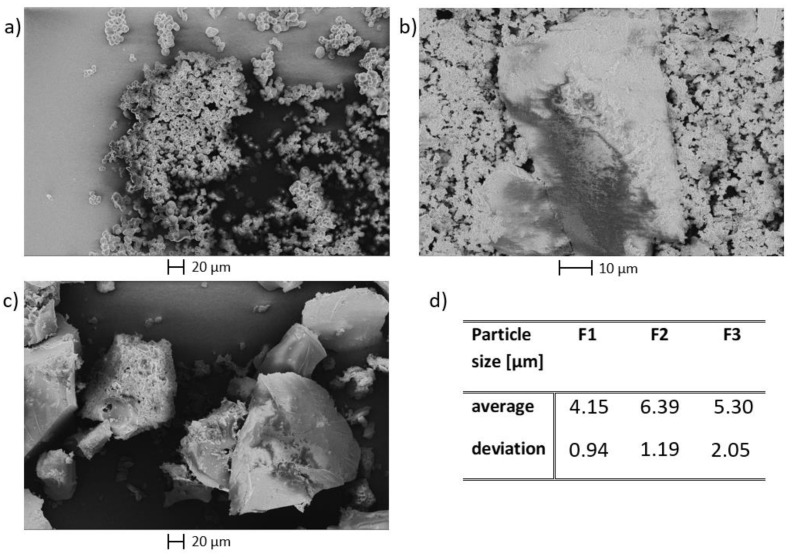
SEM micrographs of different humins: (**a**) F1, (**b**) F4, (**c**) F7, (**d**) average particle sizes of spherical humins.

With regard to the oxidative valorization of humins, their molecular structure and their particle morphology and size is important. Differences between humins based on different sugars were already examined by electron microscopy and were discussed sufficiently in a previous study [8]. Therefore, SEM micrographs were only taken of selected fructose-based humins (Figure 7), to investigate the influence of solvents and acid catalysts.

The fructose-based humins formed in water possess the typical humin structure of interconnected spheres, the main point of difference being the average particle sizes. Sulfuric acid-catalyzed humin particles were the smallest, while the p-TSA catalyzed ones were the largest, whereby the acetic acid resulted in an intermediate particle size. We, therefore, concluded that particle size was not related to the acid strength. The differences in particle size may also result from the erratic nature of humin formation as humin morphology itself varied greatly in the same reaction, as can also be seen on the micrographs [9]. This would indicate that the morphology of humins was unaffected by the acidic catalyst.

Exceptions in the morphology were the humins F4 (from EtOH/water) and F7 (from DMSO/water), which showed significant deviations from the spherical humin particles.

F4 seemed to consist of larger splinter-like fragments and a multitude of small amorphous particles were observed. F7 also featured splinter-like structures, although these had a slightly different appearance. The splinters of F7 did not appear as uniform as those of F4, a kind of fragmentation of the splinters was observed here.

Overall, the analysis showed that the solvent had the greatest influence on the structure of the humins formed. Sugar and acidic catalyst choice seemed to have a lesser influence.

### 3.2. Oxidative Conversion of Humins Using Polyoxometalate Catalysts

In the second part of our study, we investigated the potential for valorization of the synthesized humins. To this end, we chose a procedure inspired by the OxFA process [36] and previous work by Maerten et al. [29] and Raabe et al. [25]. Therefore, the humins were first dispersed in an aqueous solution containing the homogeneous polyoxometalate catalyst (H_8_PV_5_Mo_7_O_40_), and were then oxidized using molecular oxygen in a high-pressure autoclave. After reaction, the composition of the gas phase was analyzed by GC and the composition of the liquid phase was determined by HPLC. Due to the sticky nature of the used humins, the combined yield (mol% C in gas phase + mol% C in liquid phase) was used as an indicator for humin conversion.

**Figure 8:**
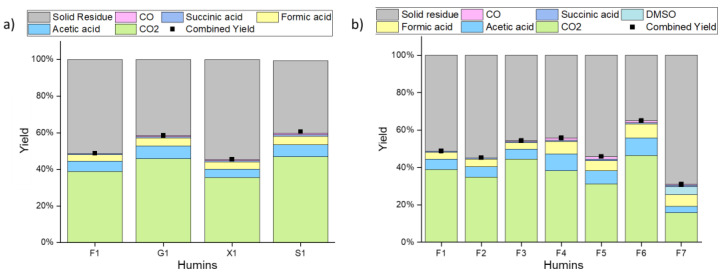
Oxidative conversion of humins using HPA-5 as a catalyst; reaction conditions: m (humin) = 75 mg, m(HPA-5) = 7.5 mg, T = 90 °C, t = 15 h, *p* = 30 bar O_2_ in 7.5 mL water as a solvent. (**a**) humins derived from different sugars (**b**) humins synthesized using different acids and solvents (determined via Equation (S3)).

Figure 8 shows two comparisons: on the left side (Figure 8a), humins derived from different sugars are compared with respect to the composition of their reaction mixtures after oxidation for 15 h. Hereby, only minor differences in terms of the product composition could be observed. Still, between 40% (S1 and G1) and 55% (X1) of the carbon remained in the solid residue after filtration. The main products for all humins were CO_2_, acetic acid, formic acid, and succinic acid. Moreover, only traces of CO could be detected in all experiments. Table 4 gives an overview of the different product yields.

On the right hand side (Figure 8b), fructose-derived humins synthesized using different acids and solvents are compared with respect to the composition of their reaction mixtures after oxidation for 15 h. Hereby, higher differences both in combined yield as well as product composition were observed. In detail, the humins synthesized in water (F1–F3) showed a clear trend that combined yield and, therefore, conversion increases with decreasing pk_a_. Therefore, using acetic acid as an acid source achieved the highest combined yield, whereby sulfuric acid showed the lowest. In terms of product composition, only minor differences could be detected (see Table 5). The humins synthesized in ethanol (F4–F6) showed a different behavior. Generally, higher carboxylic acid yields could be achieved compared to using water as a solvent (for details, see Table 5). The higher yields of acetic acid and formic acid presumably resulted from the higher degree of etherification and esterification products of ethanol, its oxidation products, and the sugar decomposition products in the reaction solution taken up in the humins. Humin F6, therefore, showed the highest product yields of all converted humins, as the larger amount of acetic acid appeared to increase the formation of the ether and ester containing compounds in the humin. The higher yields for the liquid-phase products of the humins synthesized in ethanol could be due to alcohols absorbed in the humins, as alcohols such as methanol increase liquid-phase yields in HPA-5 catalyzed syntheses and reduce the yields gained through total oxidation of biomass such as CO_2_ [50]. F5, again, showed a lower combined yield as F4 and F6. The lower O/C ratio of F5 could be a factor here, as it indicates a lower number of ether and ester bonds, although this does not explain the lower combined yield of F2 in comparison to F1 and F3. This could also be an explanation for the differences in yields between F1, G1, X1, and S1, as the humins with the highest yields (G1 and S1) showed the highest O/C ratios. The lowest combined yield of all converted humins, however, was shown by the humin F7. Interestingly, this humin showed the highest yield of carboxylic acids and, consequently, the lowest CO_2_ yield. Thioesters and -ethers were only poorly converted, while aliphatic ether and ester bonds showed a better performance.

Comparing the elemental analyses of the solids before (Tables S3–S6) with the residues after reaction (Tables S9–S11), all humins show lower carbon percentages while the oxygen percentages increased. Therefore, the oxygen to carbon ratio in the humins increased on average by a factor of 1.5. From this fact, we conclude that the residual materials have more carbonyl and hydroxy functionalities due to the catalytic oxidation. The hydrogen proportions did not change significantly. The above described trends between the humins generally remained constant.

In order to confirm which structural features of the humins were preferentially broken down during the oxidative treatment, we compared the IR spectra of the humins and their solid residues after the reaction (Figure 9 and Figures S42–S49).

The comparison of the spectra before and after the oxidation process revealed significant differences: the bands corresponding to poly substituted furan rings (795 cm^−1^, 1510 cm^−1^), aldehydes, and ethers (1090 cm^−1^, 1670 cm^−1^), and aliphatic chains (1295 cm^−1^, 1460 cm^−1^) disappeared completely. The signals belonging to unconjugated double bonds (1020 cm^−1^) and esters (1700 cm^−1^) were much weaker. The band corresponding to carbonyl conjugated C=C double bonds (1600 cm^−1^), on the other hand, became more intense and shifted to slightly lower wavenumbers. Together, this indicates that ethers and esters were converted very well in the HPA-5 catalyzed oxidation.

This also explains the decrease in the signals corresponding to substituted furan rings and aliphatic chains. If the aliphatic chains were linked via ether or ester bonds, they would consequently also be broken down. The most interesting observation here was the disappearance of the vibration band at 795 cm^−1^, indicating substituted furan rings, which apparently were no longer present after the conversion. Therefore, it is reasonable to assume that this signal corresponded to furan rings that were connected through by ester or ether bonds which were broken down. This also indicates that in the structure of the humins that were produced in the DMSO-containing solution, these furan esters/ethers occurred little or not at all, explaining the lower conversion of F7. However, this did not explain the apparent disappearance of the band corresponding to C=C double bond vibration of substituted furans (1510 cm^−1^). A disappearance of this band could normally indicate a splitting of the furan rings, but since all other signals of the furan rings were generally unchanged, it was assumed that the signal was swallowed up by the more intense signal of the carbonyl conjugated double bonds at 1600 cm^−1^. The increase in this vibration band was due to the oxidation of the remaining insoluble humin residues. Together with the results from elemental analysis, which showed that the O content increased, this indicates that alkyl groups or furan rings which could not be cleaved were oxidized to form either double bonds or ketones. This would drastically increase the number of double bonds conjugated to a carbonyl compound in the humins, explaining the much more intense signal at 1600 cm^−1^_,_ and would simultaneously explain the loss in intensity of the band corresponding to unconjugated C=C double bonds (1020 cm^−1^). In addition to these changes in the vibration bands, some entirely new peaks could be found in the range between 1000 and 1100 cm^−1^. These signals most likely belonged to the catalyst HPA-5, as IR spectra of the catalyst showed asymmetric stretching vibrations of the P-O bonds in the range from 1041 to 1066 cm^−1^. The vibrational band at 965 cm^−1^ could also have been influenced by the catalyst, since HPA-5 showed an intensive signal from the M=O bond at this wavenumber [51].

**Figure 10:**
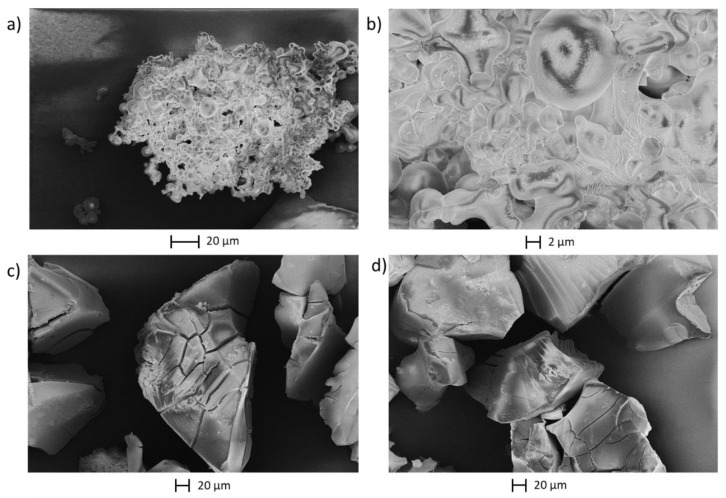
SEM micrographs after the oxidative catalysis with HPA-5 (**a**) F1 (**b**) F1 zoomed in (**c**) F4 (**d**) F7.

As morphological differences between the humins before the catalytic oxidation were primarily observed between humins from different solvents, F1, F4, and F7 were further studied by electron microscopy after conversion (Figure 10).

The images of F1 after the reaction (Figure 10a,b) show that the material was not comprised out of spherical particles anymore. Instead, they were fused to an amorphous cluster, as if they had melted. The particles of F4 and F7, on the other hand, appeared mostly unchanged after the oxidative treatment. The amorphous particles visible in Figure 7b,c completely disappeared from the structure and only the splinter-like particles were left, but here, they no longer had a rough surface. It seems as if primarily agglomerated oligomers were converted by the oxidative catalysis but the polymer core structures were not affected.

## 4. Conclusions

In summary, the formation of humins was studied in 28 different substrate/solvent/acid combinations. Differences between the liquid and solid phase of each experiment were analyzed by selected analytical methods. We found that the choice of solvent had the greatest influence on the molecular structure and the yield of the synthesized humins. By comparing the MALDI-TOF MS spectra and infrared spectra of the many different humins, we were able to identify various functional groups such as furan rings, ethers, and carbonyl moieties. Furthermore, we were able to identify three distinct structural fragments, with two of these fragments being found in each of the analyzed humins.

With respect to the valorization of the humins, the polyoxometalate (H_8_PV_5_Mo_7_O_40_) catalyzed oxidation was partially successful. In most cases, half of the humin mass could be converted. Unfortunately, the main product was CO_2_, which only possessed limited economical applications. However, more valuable products such as acetic acid, formic acid, and succinic acid could also be obtained. Humin F6, which was formed from fructose in EtOH/H_2_O using acetic acid, was converted particularly well, yielding larger quantities of acetic acid and formic acid than all other humins. From the correlation between humin structure and conversion, we conclude that ether and ester groups were converted very well by catalytic oxidation, while thioester and ether groups were only poorly converted. The furan-containing core structure of the humins remained mostly unaffected by the catalytic oxidative treatment.

Overall, the yield in the humin synthesis was the lowest for the humins with acetic acid as the catalyst and the ethanol/water solution as the solvent and F6 showed the greatest potential for valorization. Therefore, we suggest that the EtOH/H_2_O/acetic acid system is the ideal choice for gaining the maximum value from sugars. These findings could potentially be used to reduce the formation of humins in a sustainable synthesis of levulinic acid and, furthermore, to convert the low amount of unavoidable humins into valuable substances.

## Figures and Tables

**Figure 9:**
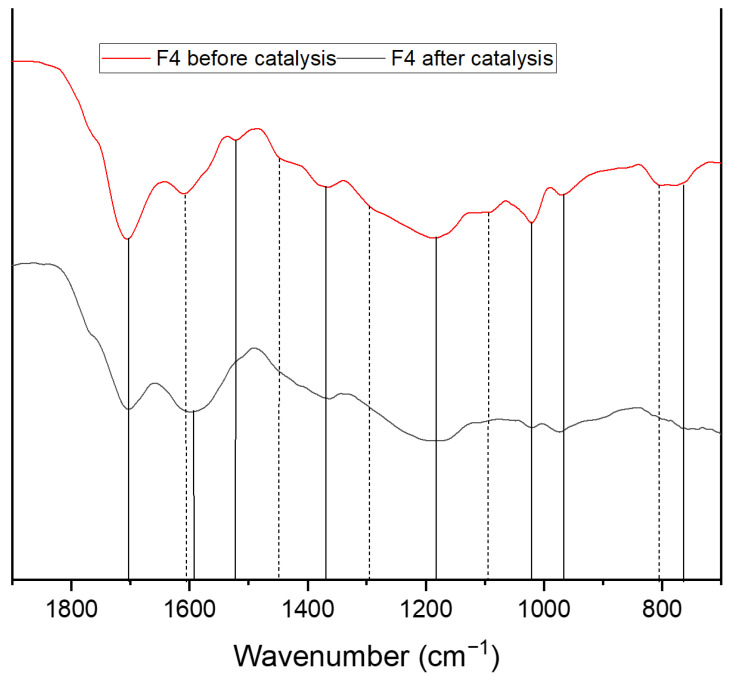
IR-spectra of F4 before and after the oxidative catalysis with HPA-5.

**Table 1:** Matrix of the humin formation experiments.

Humin	H_2_O			EtOH/H_2_O			DMSO/H_2_O
Designation	H_2_SO_4_	p-TSA	AA	H_2_SO_4_	p-TSA	AA	H_2_SO_4_
Fructose	F1	F2	F3	F4	F5	F6	F7
Glucose	G1	G2	G3	G4	G5	G6	G7
Xylose	X1	X2	X3	X4	X5	X6	X7
Sucrose	S1	S2	S3	S4	S5	S6	S7

**Table 2:** Sugar conversion in humin formation experiments.

	H_2_O			EtOH/H_2_O			DMSO/H_2_O
X (%)	H_2_SO_4_^a^	p-TSA ^a^	AA ^a^	H_2_SO_4_^b^	p-TSA ^b^	AA ^b^	H_2_SO_4_^b^
Fructose	100	100	100	100	100	100	100
Glucose	90	74	69	100	100	100	100
Xylose	100	100	100	100	100	100	100
Sucrose	90.5	94	84	100	100	100	100

Experimental Conditions: 10-fold reaction system, 180 °C, 45 bar (1:4 O_2_/N_2_), 5 mL substrate mixture with 1 M sugar, pH 2, 6 h. ^a^ Determined through HPLC (Equation (S1)). ^b^ Determined by ^1^H-NMR.

**Table 3:** Assignment of IR-spectra bands.

**Wavenumber [cm^−1^]**	**Assignment**
750 + 795 965	C-H Out of plane vibration substituted furan ring C-H vibration furan ring
1020	C=C stretch vibration
1090	C-O-C ether vibration
1160 + 1200	C-O-C deformation vibration furan ring
1295	C-H rocking vibration
1360	C-C framework vibration (furan) C6 sugars
1395	C-C framework vibration (furan) C5 sugars
1420	C=S stretch
1460	C-H aliphatic chain vibration
1510	C=C vibration aromatic double bonds of poly substituted furans
1600	C=C stretch vibration conjugated with carbonyl
1670	C=O carbyonyl, aldehyde vibrations
1700	C=O stretch of acids, aldehydes and ketons
1775	C-S Thioester

**Table 4:** Product yields using different sugar-derived humins; reaction conditions: m (humin) = 75 mg, m(HPA-5) = 7.5 mg, T = 90 °C, t = 15 h, *p* = 30 bar O_2_ in 7.5 mL water as a solvent.

(a) Product Yields	F1	G1	X1	S1
CO_2_	39%	46%	36%	47%
Acetic acid	6%	7%	5%	7%
Formic acid	4%	4%	4%	5%
Succinic acid	1%	1%	1%	1%
Combined Yield	49%	59%	45%	61%

**Table 5:** Product yields using fructose-derived humins using different acids and solvents; reaction conditions: m (humin) = 75 mg, m(HPA-5) = 7.5 mg, T = 90 °C, t = 15 h, *p* = 30 bar O_2_ in 7.5 mL solvent.

(a) Product Yields	F1	F2	F3	F4	F5	F6	F7
CO_2_	39%	35%	44%	38%	31%	46%	16%
Acetic acid	6%	6%	5%	9%	7%	9%	3%
Formic acid	4%	4%	3%	7%	5%	7%	6%
Succinic acid	1%	1%	1%	1%	1%	1%	1%
Combined Yield	52%	61%	65%	74%	53%	82%	73%

## Data Availability

Data is provided in the Supplementary Information File.

## Supplementary Materials

The following supporting information can be downloaded at: https://www.mdpi.com/article/10.3390/ma16072864/s1.
