# Supplementary material for: Investigation of the Formation, Characterization, and Oxidative Catalytic Valorization of Humins
Source: Materials (Basel). 2023 Apr 4;16(7):2864. doi: 10.3390/ma16072864 (PMC10095678; doi:10.3390/ma16072864)
Supplement: Supplementary file 1 [file materials-16-02864-s001.zip › materials-2286656-supplementary.pdf]

Supporting Information

Humin Synthesis

NMR spectra:

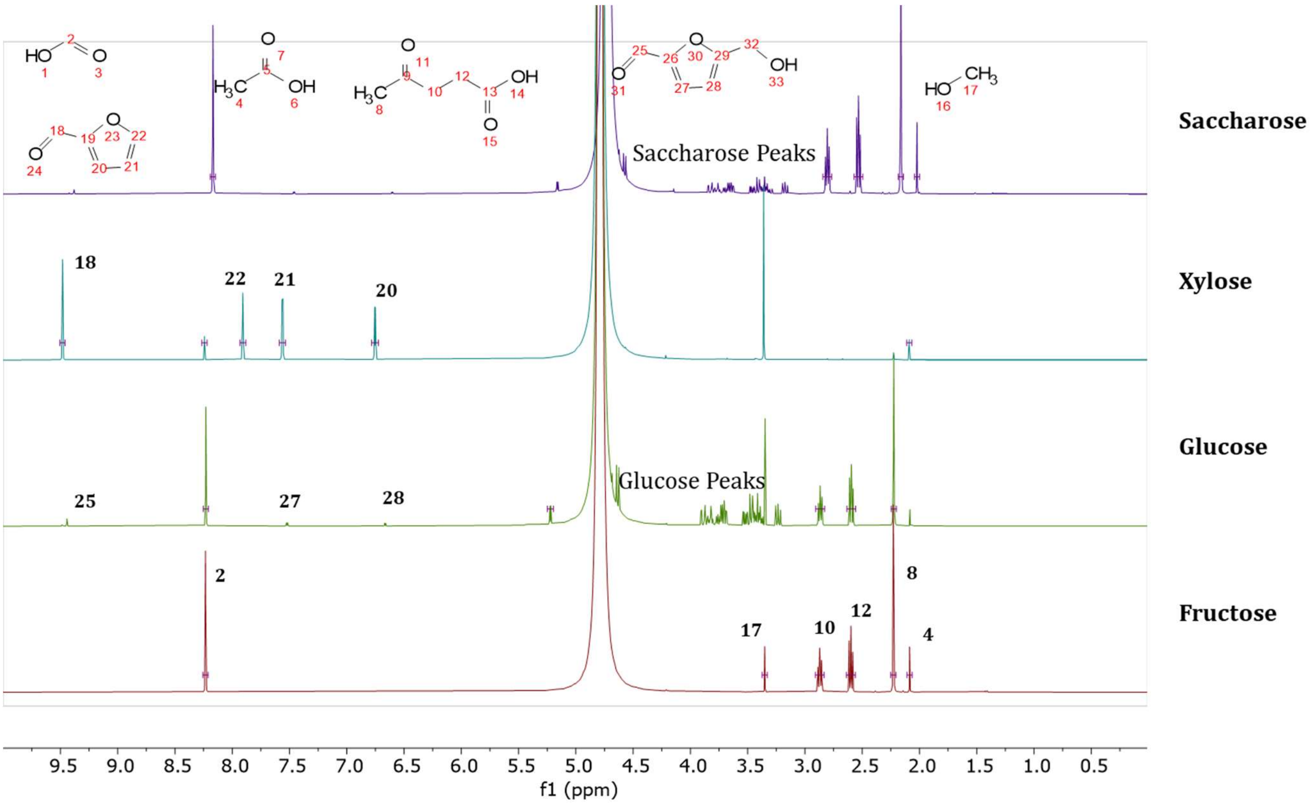

Figure S1:^{1}H-NMR spectra of humins synthesized in sulfuric acid and water taken in D_2O

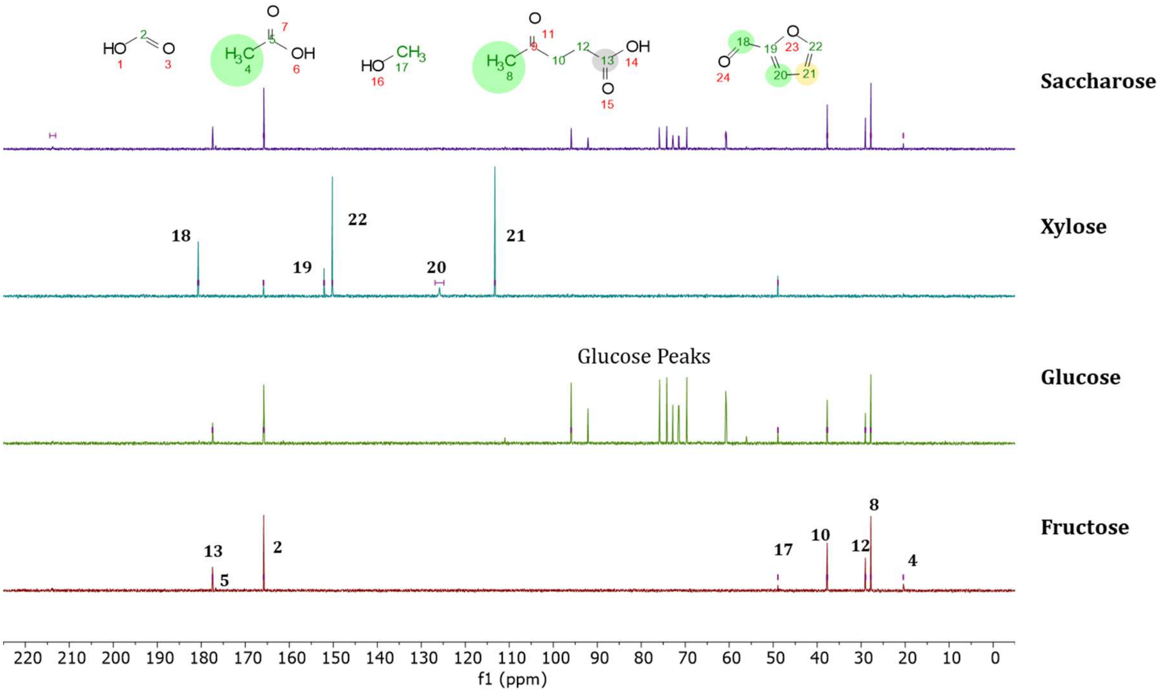

Figure S2: ^{13}C-NMR spectra of humin synthesized in sulfuric acid and water taken in D_2O

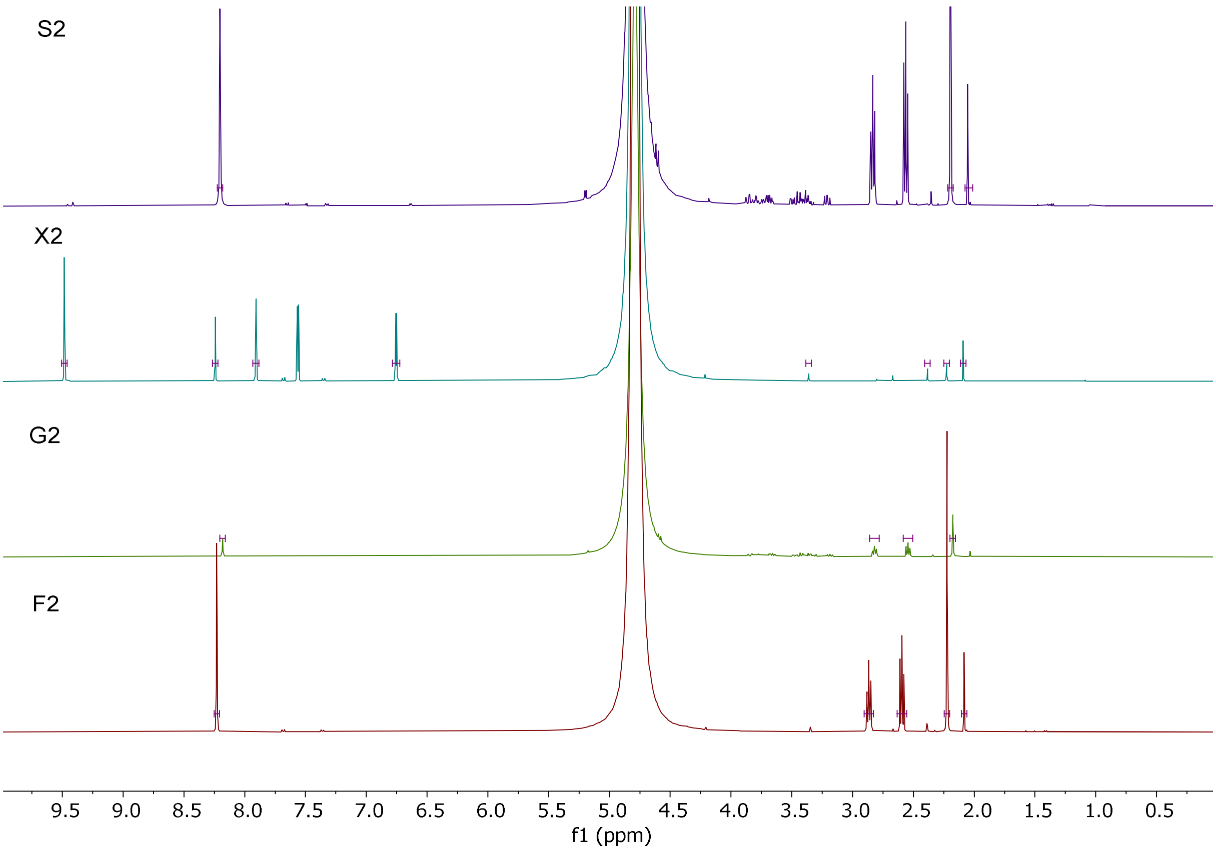

Figure S3:^{1}H-NMR spectra of humins synthesized in para toluenesulfonic acid and water taken in D_2O

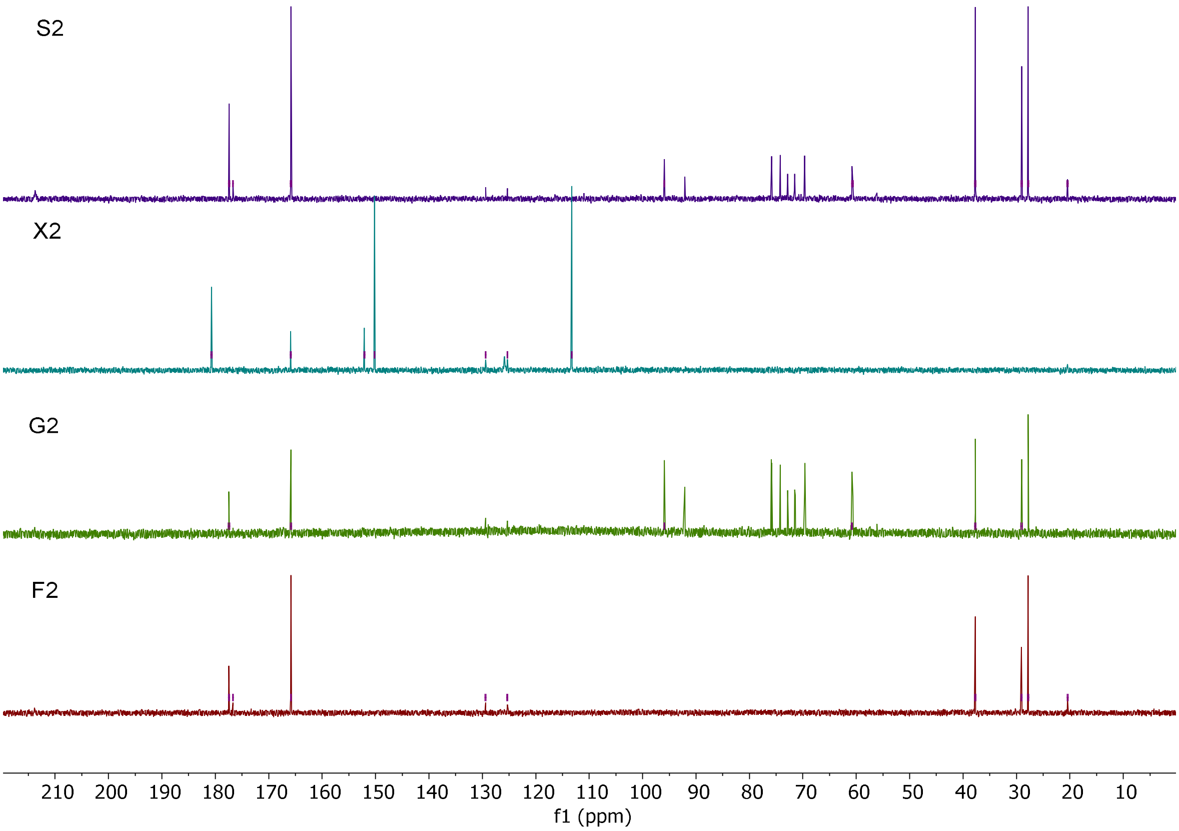

Figure S4: ^{13}C-NMR spectra of humins synthesized in para toluenesulfonic acid and water taken in D_2O

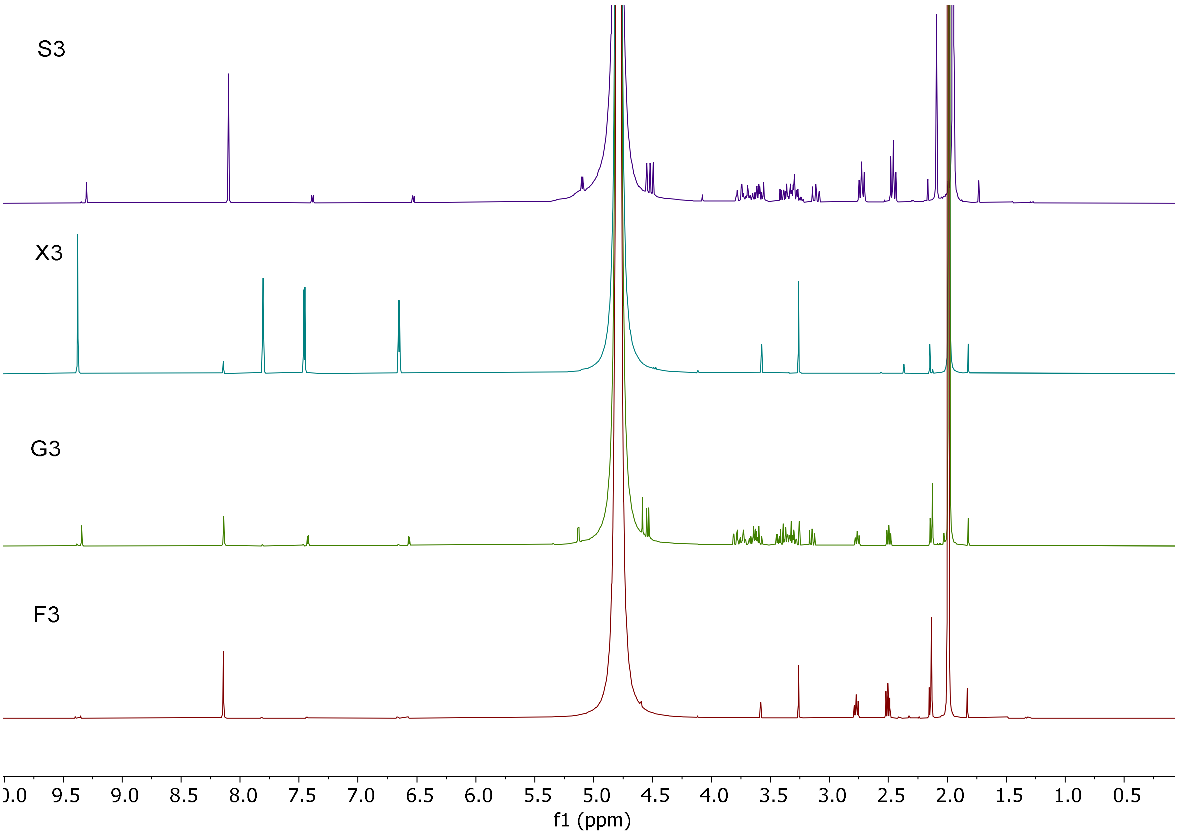

Figure S5:^{1}H-NMR spectra of humins synthesized in acetic acid and water taken in D_2O

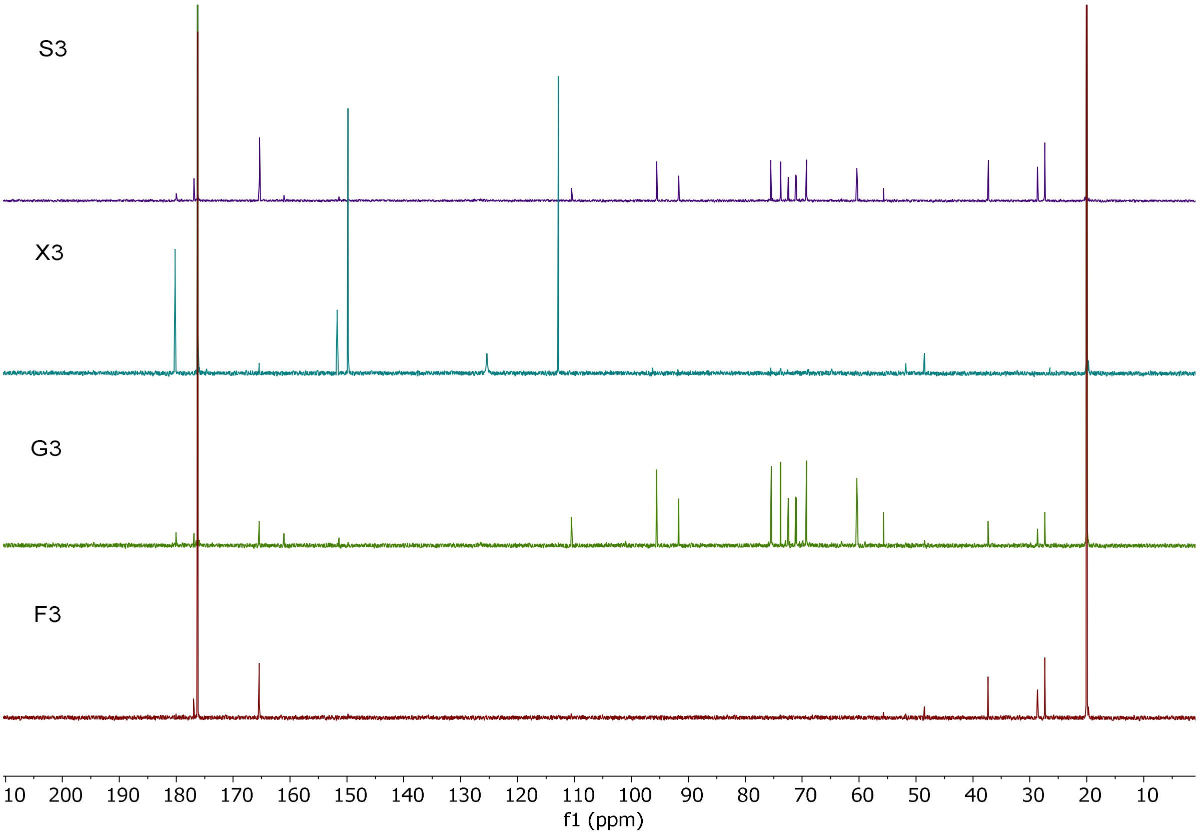

Figure S6: ^{13}C-NMR spectra of humins synthesized in acetic acid and water taken in D_2O

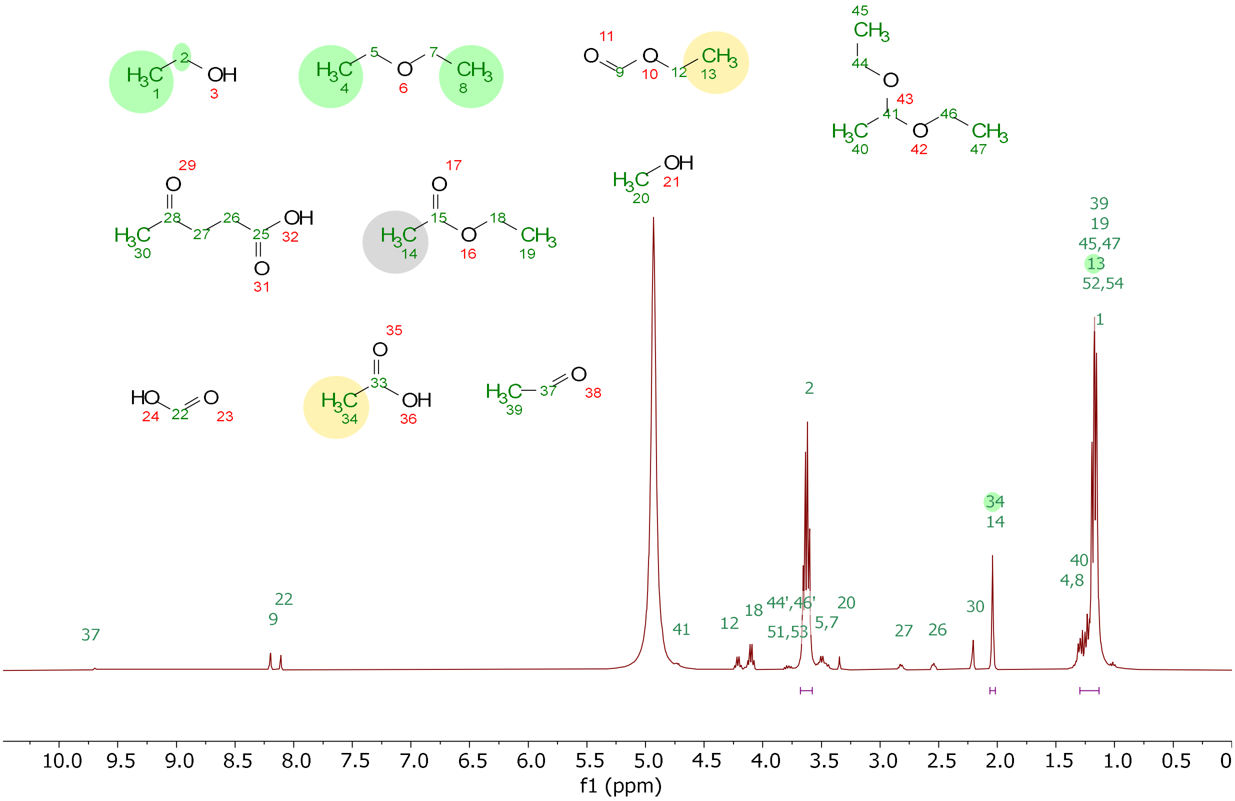

Figure S7:^{1}H-NMR spectra of F4 taken in D_2O

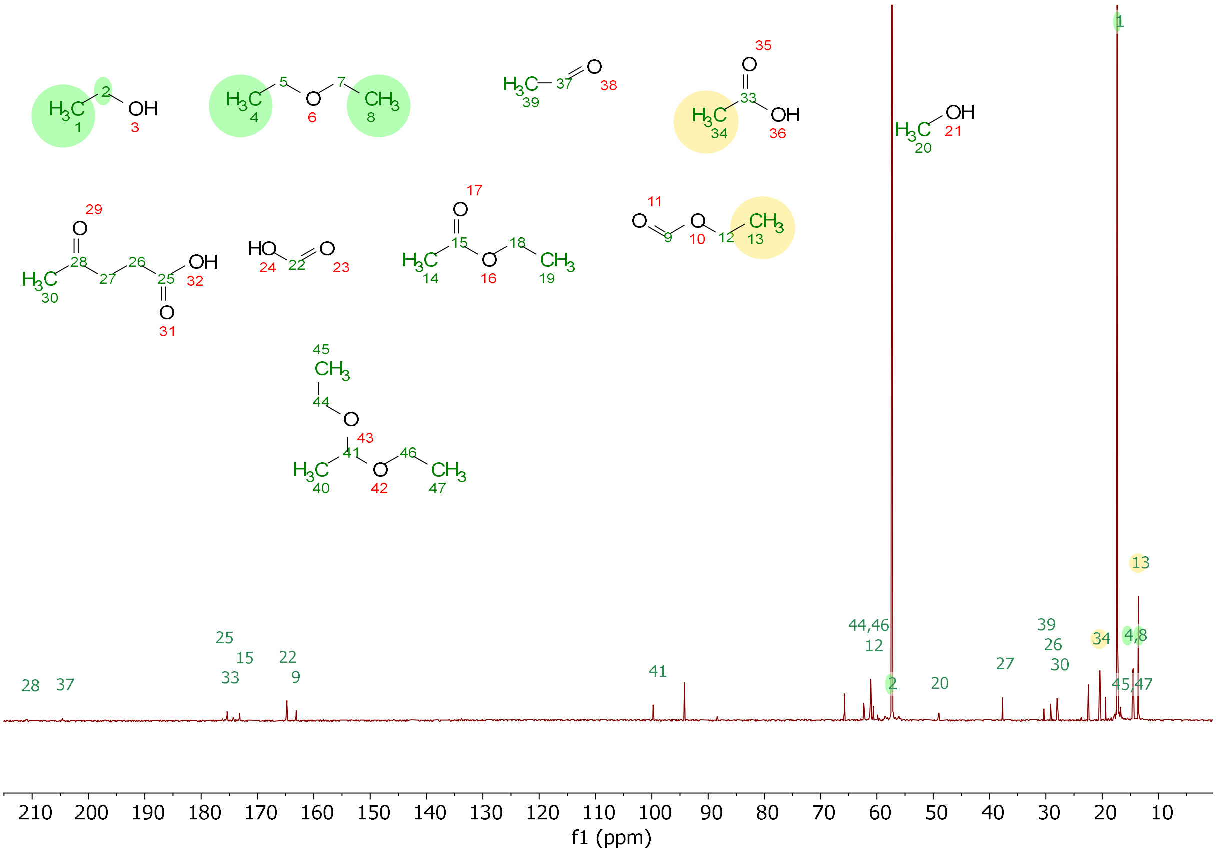

Figure S8: ^{13}C-NMR spectra of F4 taken in D_2O

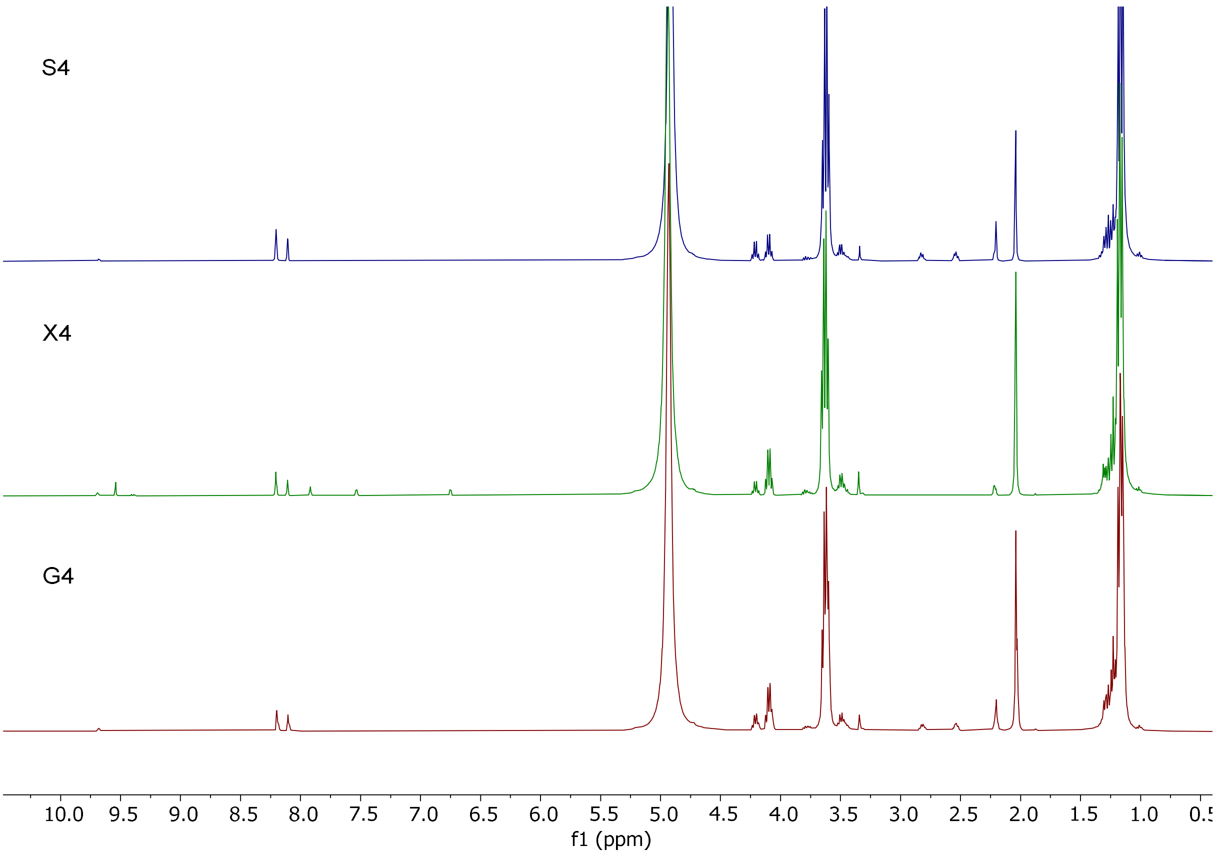

Figure S9: ^{1}H-NMR spectra of humins synthesized in sulfuric acid and water/ethanol

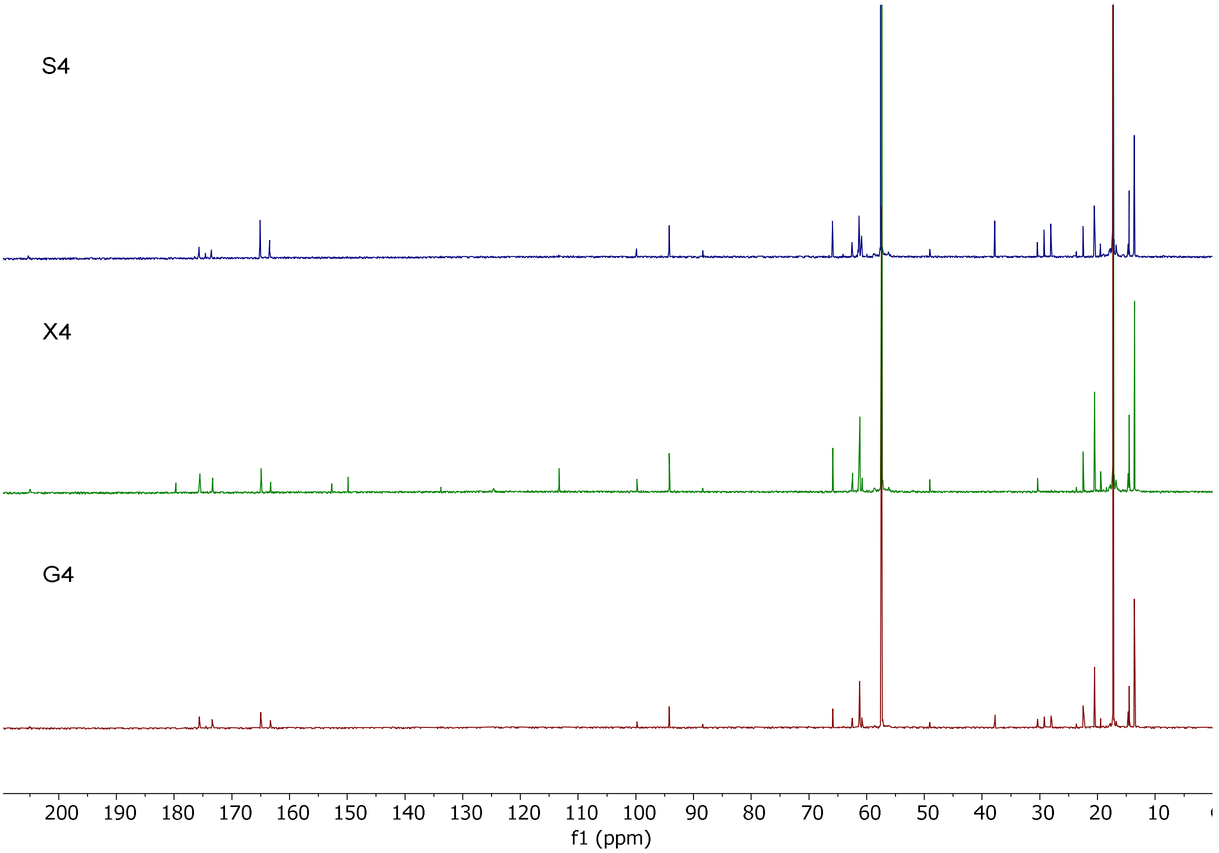

Figure S10: ^{13}C-NMR spectra of humins synthesized in sulfuric acid and water/ethanol taken in D_2O

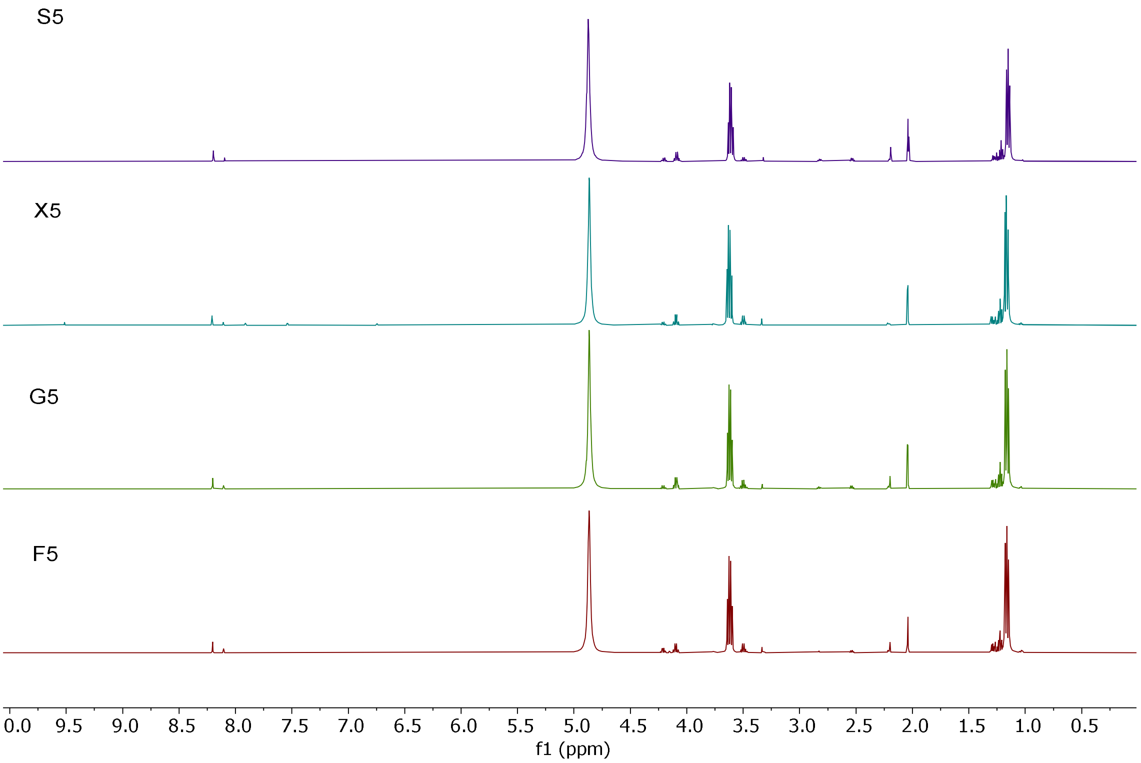

Figure S11:^{1}H-NMR spectra of humins synthesized in para toluene sulfonic acid and water/ethanol taken in D_2O

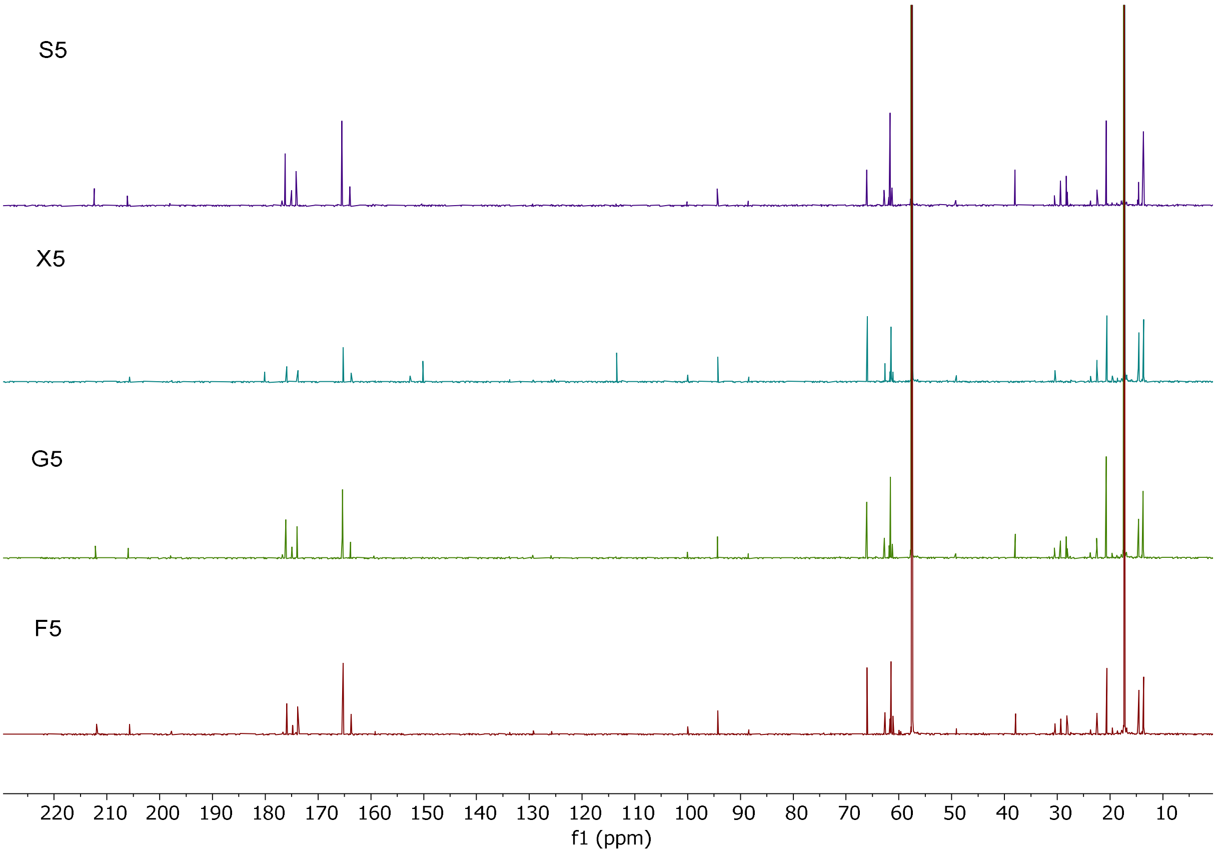

Figure S12: ^{13}C-NMR spectra of humins synthesized in para toluenesulfonic acid and water/ethanol taken in D_2O

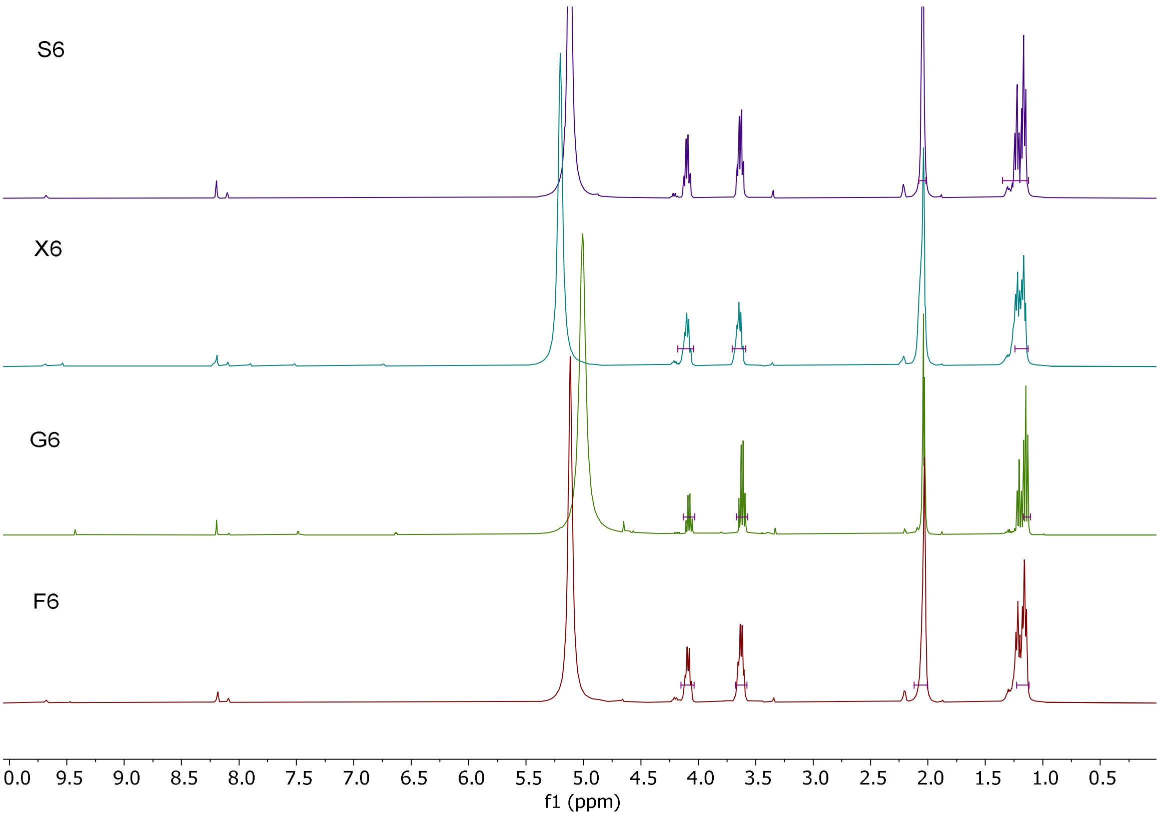

Figure S13:^{1}H-NMR spectra of humins synthesized in acetic acid and water/ethanol taken in D_2O

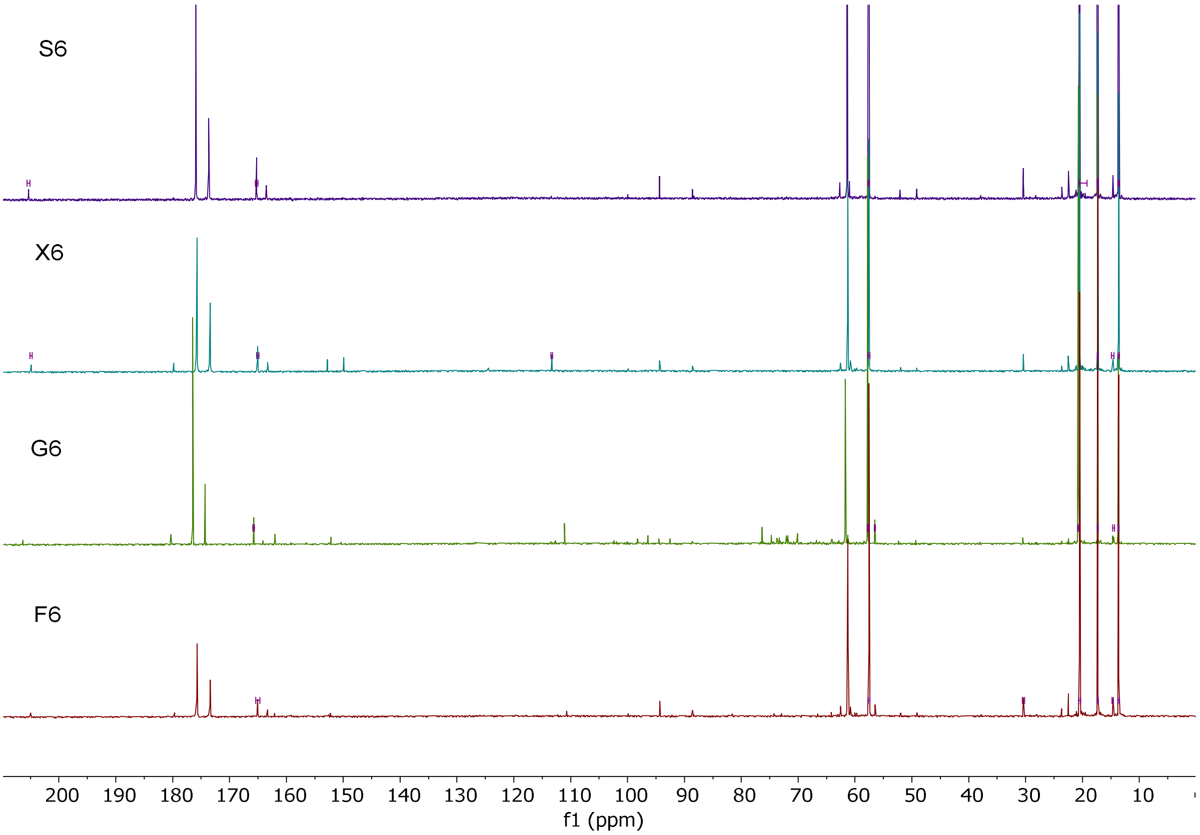

Figure S14: ^{13}C-NMR spectra of humins synthesized in acetic acid and water/ethanol taken in D_2O

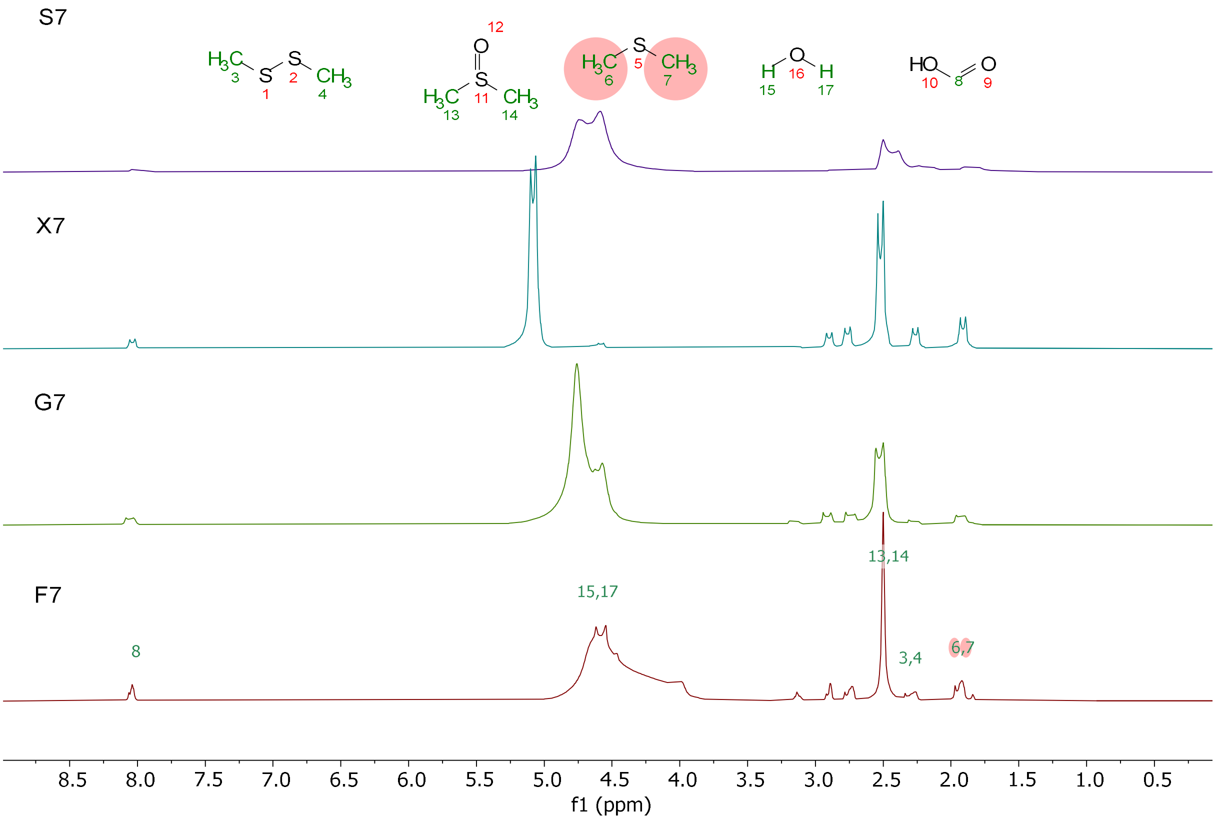

Figure S15:^{1}H-NMR spectra of humins synthesized in sulfuric acid and water/dimethyl sulfoxide taken in DMSO-d_6

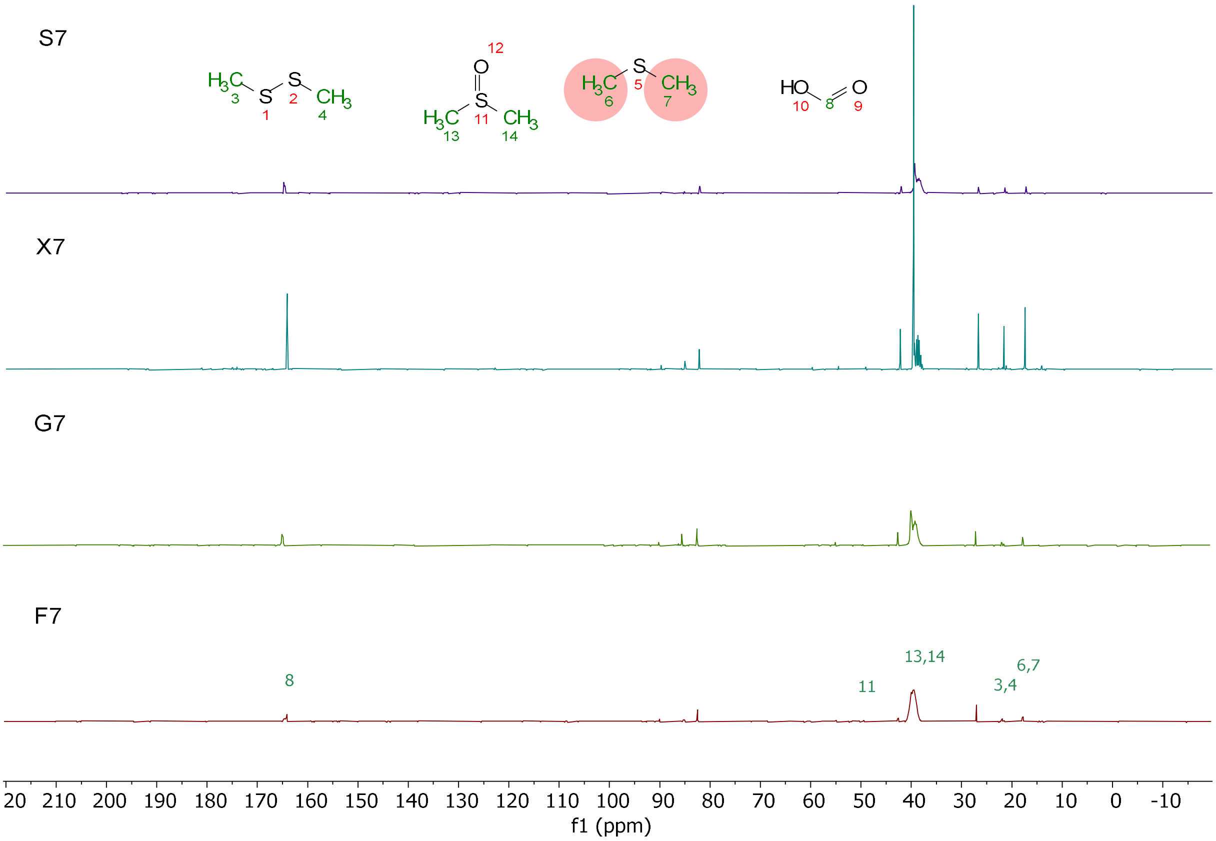

Figure S16: ^{13}C-NMR spectra of humins synthesized in sulfuric acid and water/dimethyl sulfoxide taken in DMSO-d_6

Elemental analysis:

Table S1: Theoretical elemental compositions of sugars used for humin synthesis

Elements	Fructose	Glucose	Xylose	Sucrose
H	6,71	6,71	6,71	6,48
C	40	40	40	42,11
O	53,28	53,28	53,28	51,41
H/C	2,01	2,01	2,01	1,85
O/C	1,00	1,00	1,00	0,92

Table S2: CHSO of humin synthesized in sulfuric acid and water

Elements	F1	G1	X1	S1
H	3.875	4.105	4.01	4.12
C	63.54	63.34	64.66	63.795
O	31.3	31.245	30.425	31.625
S	0	0	0	0
Sum	98.715	98.69	99.095	99.54
H/C	0.73	0.78	0.74	0.77
H/O	0.37	0.37	0.35	0.37

Table S3: CHSO of fructose humins

Elements	F1	F2	F3	F4	F5	F6	F7
H	3.875	3.915	3.7	4.565	4.905	4.165	4.185
C	63.54	62.595	62.985	65.175	67.26	61.7	58.69
O	31.3	32.14	32.225	29.6	26.48	32.78	26.015
S	0	0	0	0	0	0	9.76
Sum	98.715	98.65	98.91	99.34	98.645	98.645	98.65
H/C	0.73	0.75	0.70	0.84	0.88	0.81	0.86
O/C	0.37	0.39	0.38	0.34	0.30	0.40	0.33

Table S4: CHSO of glucose humins

Elements	G1	G2	G3	G4	G5	G6	G7
H	4.11	4.00	3.95	4.83	5.17	3.88	4.12
C	63.34	62.62	62.77	65.87	68.09	59.36	59.00
O	31.25	32.23	32.40	28.34	25.66	34.38	26.85
S	0.00	0.00	0.00	0.00	0.00	0.00	8.99
Sum	98.69	98.84	99.11	99.04	98.91	97.61	98.95
H/C	0.78	0.77	0.76	0.88	0.91	0.78	0.84

**O/C**	0.37	0.39	0.39	0.32	0.28	0.43	0.34

Table S5: CHSO of xylose humins

Elements	X1	X2	X3	X4	X5	X6	X7
**H**	4.01	3.71	4.01	4.70	4.79	4.03	4.06
**C**	64.66	63.87	63.92	66.51	68.29	61.42	58.92
**O**	30.43	31.17	31.24	28.08	25.50	29.26	27.28
**S**	0.00	0.00	0.00	0.00	0.00	0.00	8.42
**Sum**	99.10	98.75	99.16	99.28	98.57	98.91	98.66
**H/C**	0.74	0.70	0.75	0.85	0.84	0.79	0.83
**O/C**	0.35	0.37	0.37	0.32	0.28	0.36	0.35

Table S6: CHSO of sucrose humins

Elements	S1	S2	S3	S4	S5	S6	S7
**H**	4.12	4.13	3.87	4.50	4.82	3.88	4.27
**C**	63.80	63.47	62.98	64.77	67.03	59.31	61.19
**O**	31.63	31.15	32.15	29.86	27.49	34.39	26.54
**S**	0.00	0.00	0.00	0.00	0.00	0.00	6.64
**Sum**	99.54	98.74	98.99	99.13	99.34	97.58	98.63
**H/C**	0.77	0.78	0.74	0.83	0.86	0.79	0.84
**O/C**	0.37	0.37	0.38	0.35	0.31	0.43	0.33

IR-spectra:

Table S7: Assignment of IR-bands

Wavenumber [cm^{-1}]	Assignment
**750+795**	C-H Out of plane vibration substituted furan ring
**965**	C-H vibration furan ring
**1020**	C=C stretch vibration
**1090**	C-O-C ether vibration
**1160+1200**	C-O-C deformation vibration furan ring
**1295**	C-H rocking vibration
**1360**	C-C framework vibration (furan) C6 sugars
**1395**	C-C framework vibration (furan) C5 sugars
**1420**	C=S stretch
**1460**	C-H aliphatic chain vibration
**1510**	C=C vibration aromatic double bonds of poly substituted furans
**1600**	C=C stretch vibration conjugated with carbonyl
**1670**	C=O carbyonyl, aldehyde vibrations
**1700**	C=O stretch of acids, aldehydes and ketons
**1775**	C-S Thioester

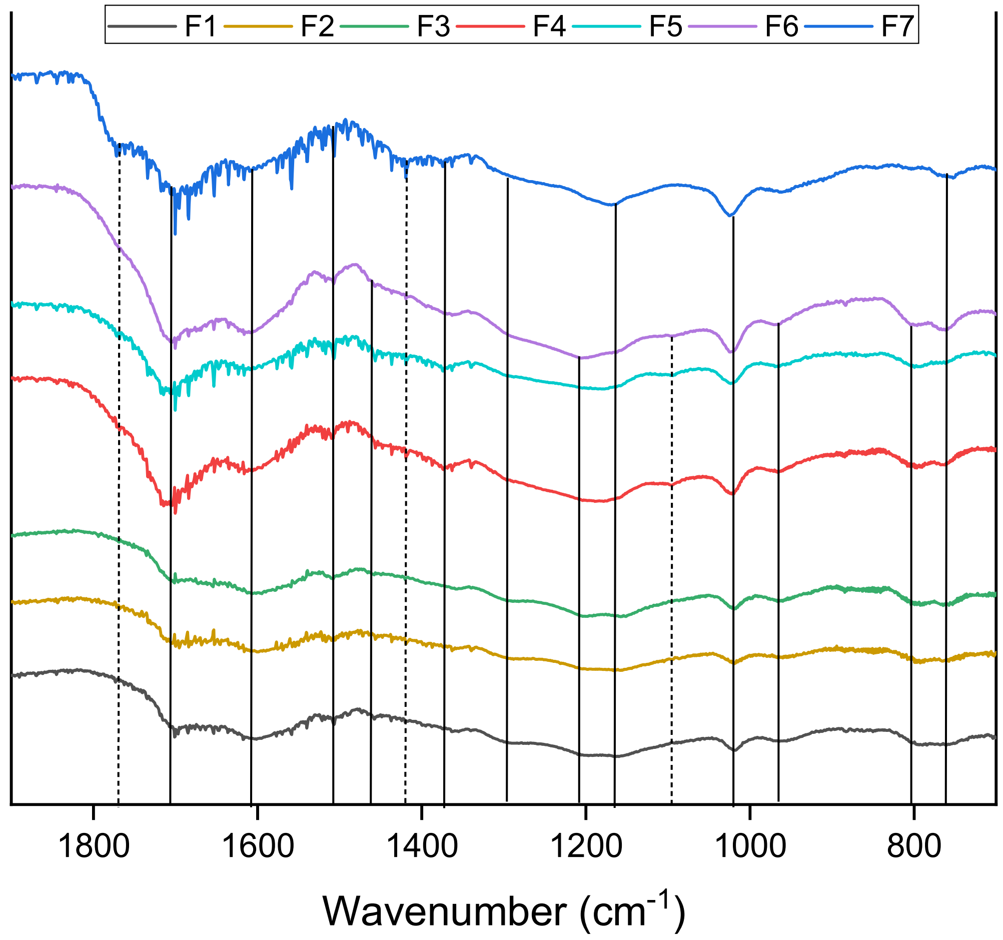

Figure S17: IR spectra of fructose humins

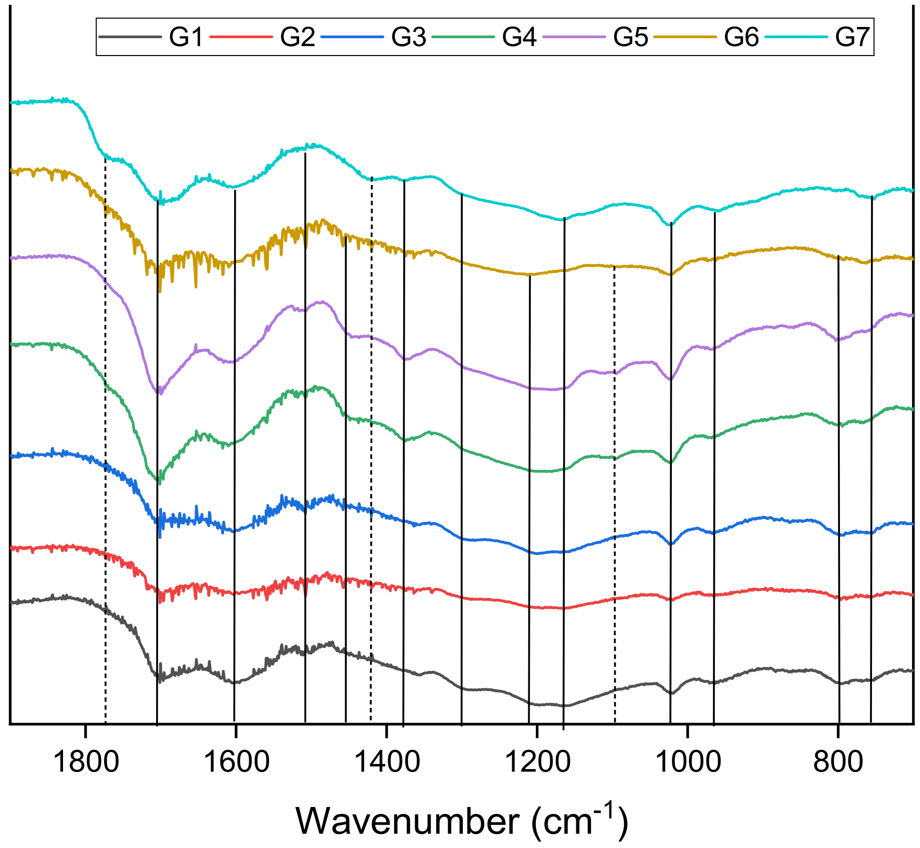

Figure S18: IR spectra of glucose humins

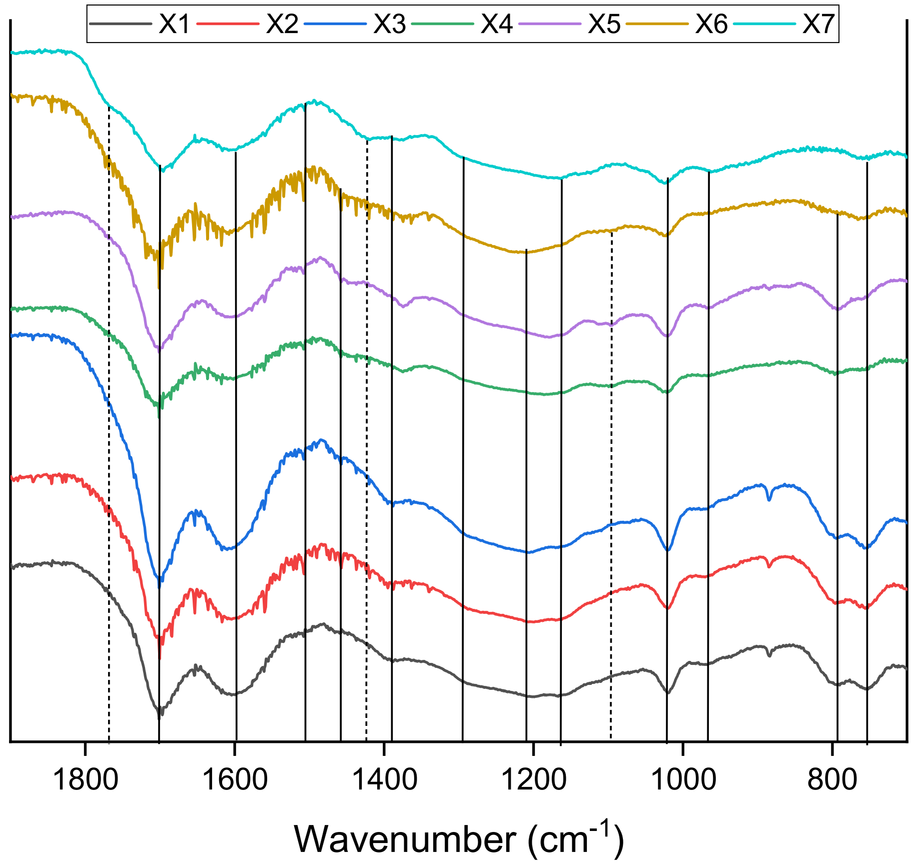

Figure S19: IR spectra of xylose humins

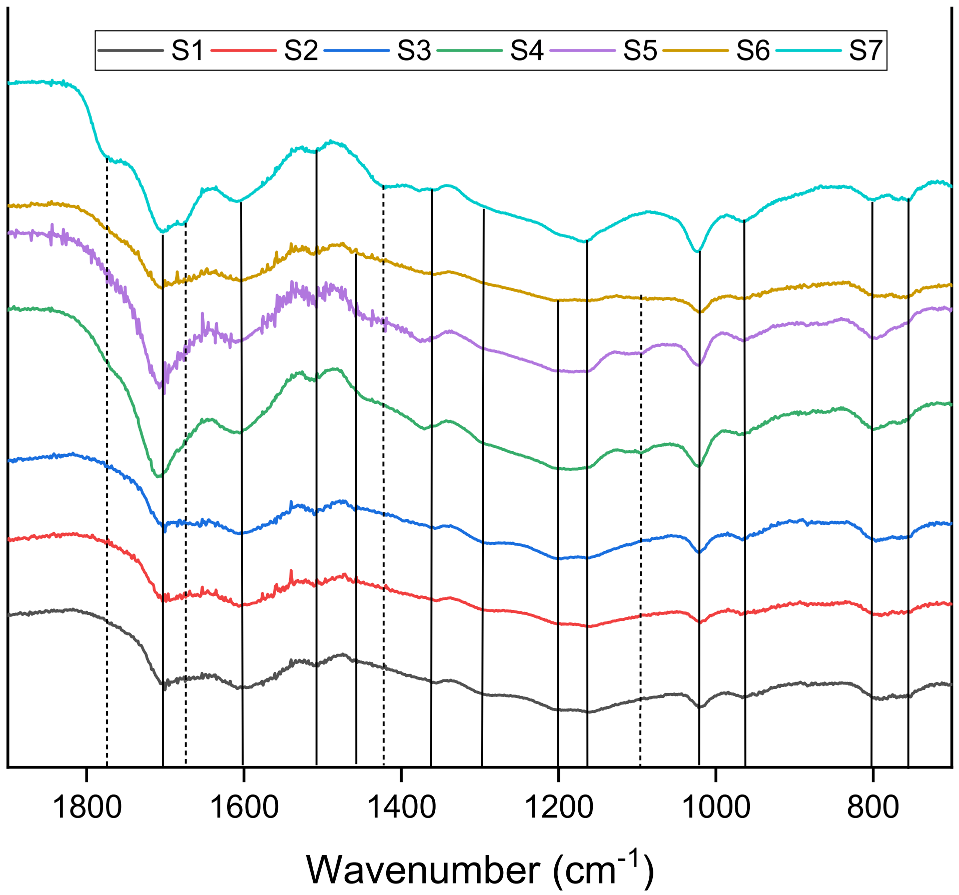

Figure S20: IR spectra of sucrose humins

MALDI-TOF MS spectra:

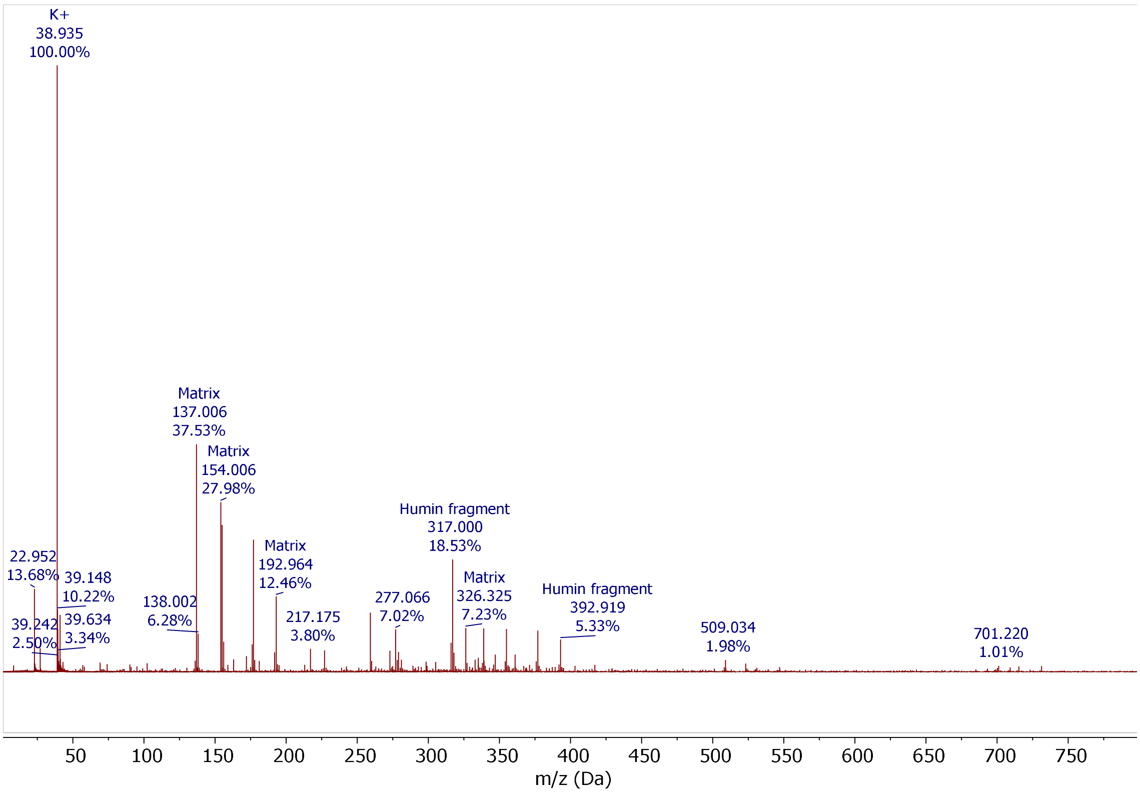

Figure S21: MALDI-TOF-MS spectrum of F1

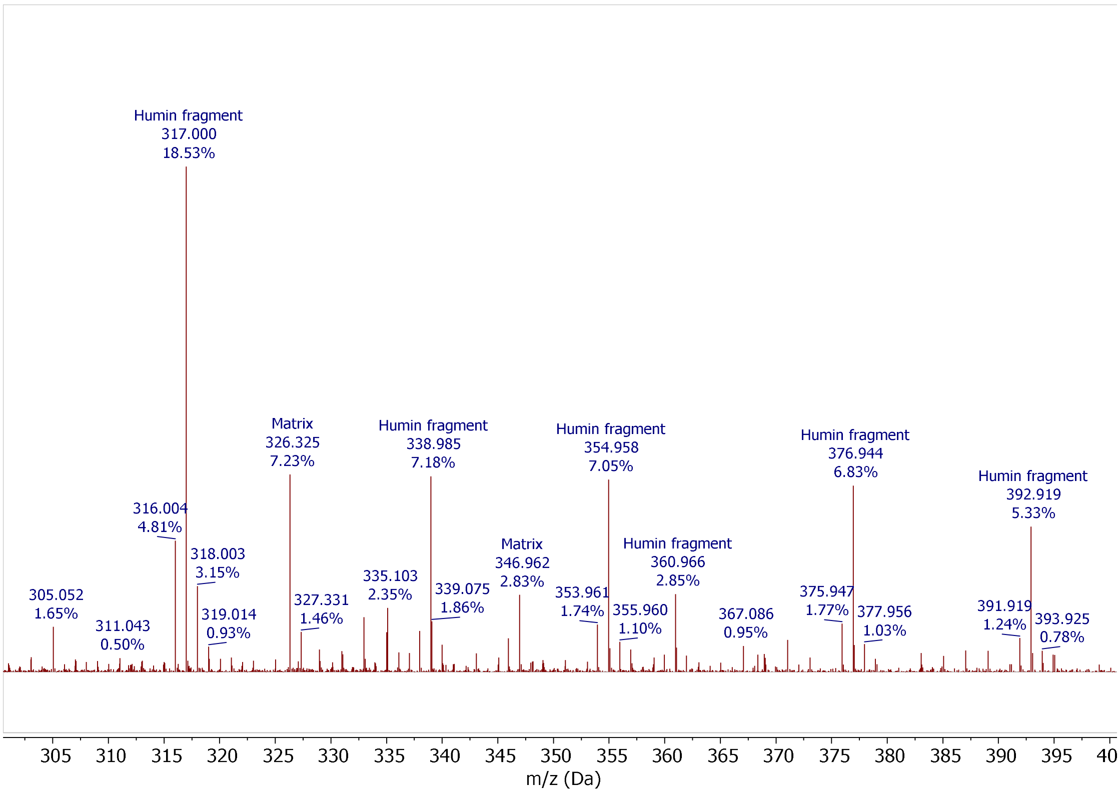

Figure S22: MALDI-TOF-MS spectrum of F1 zoomed in

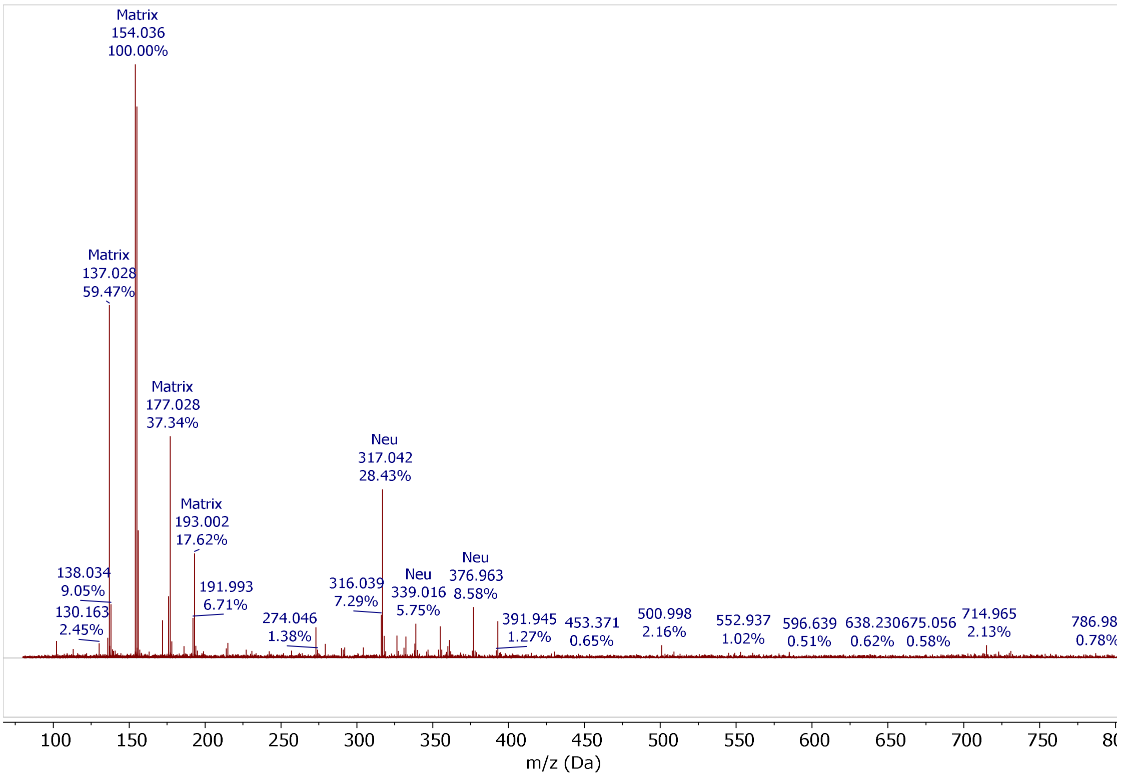

Figure S23: MALDI-TOF-MS spectrum of F2

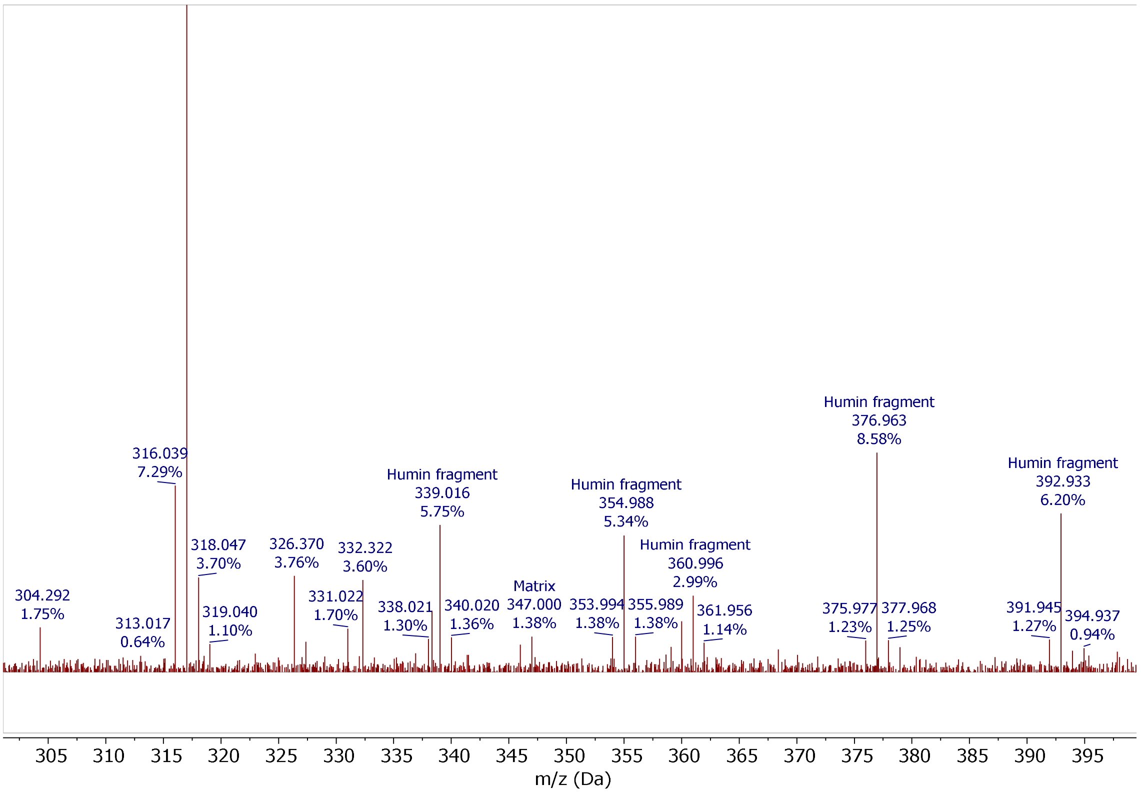

Figure S24: MALDI-TOF-MS spectrum of F2 zoomed in

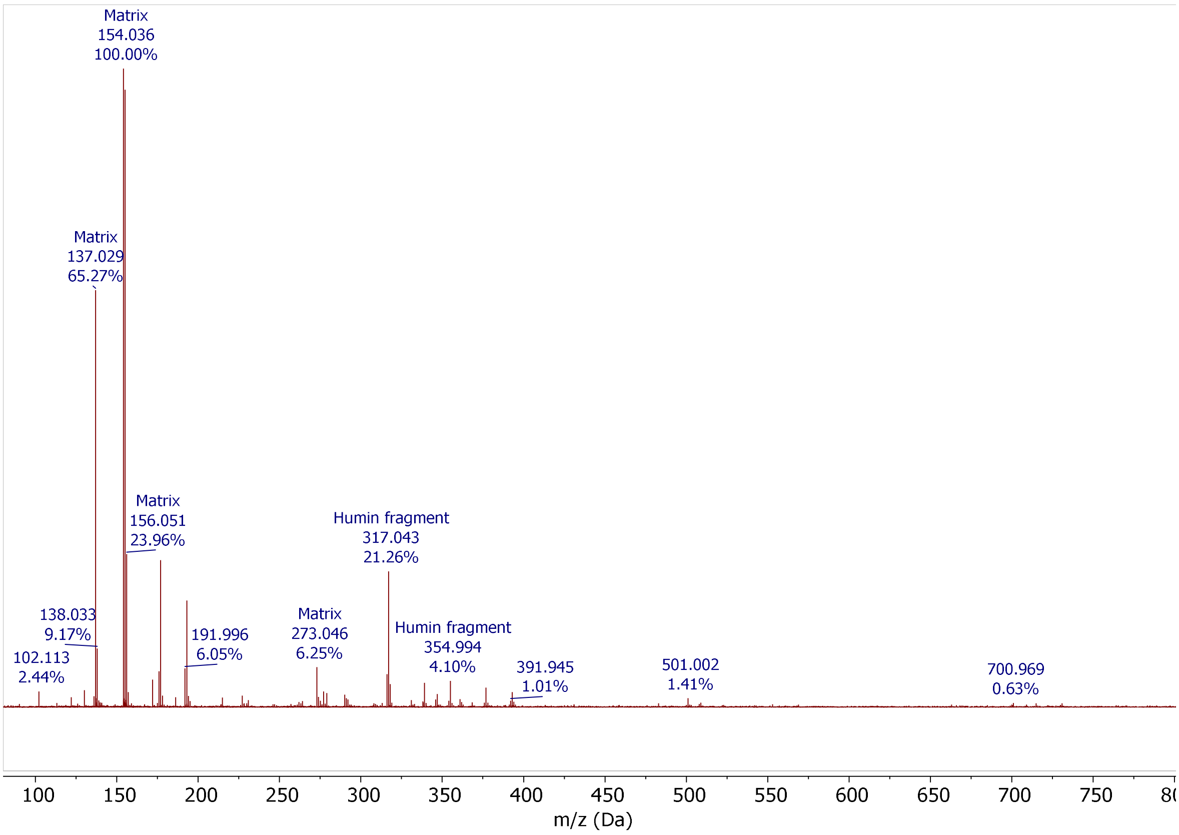

Figure S25: MALDI-TOF-MS spectrum of F3

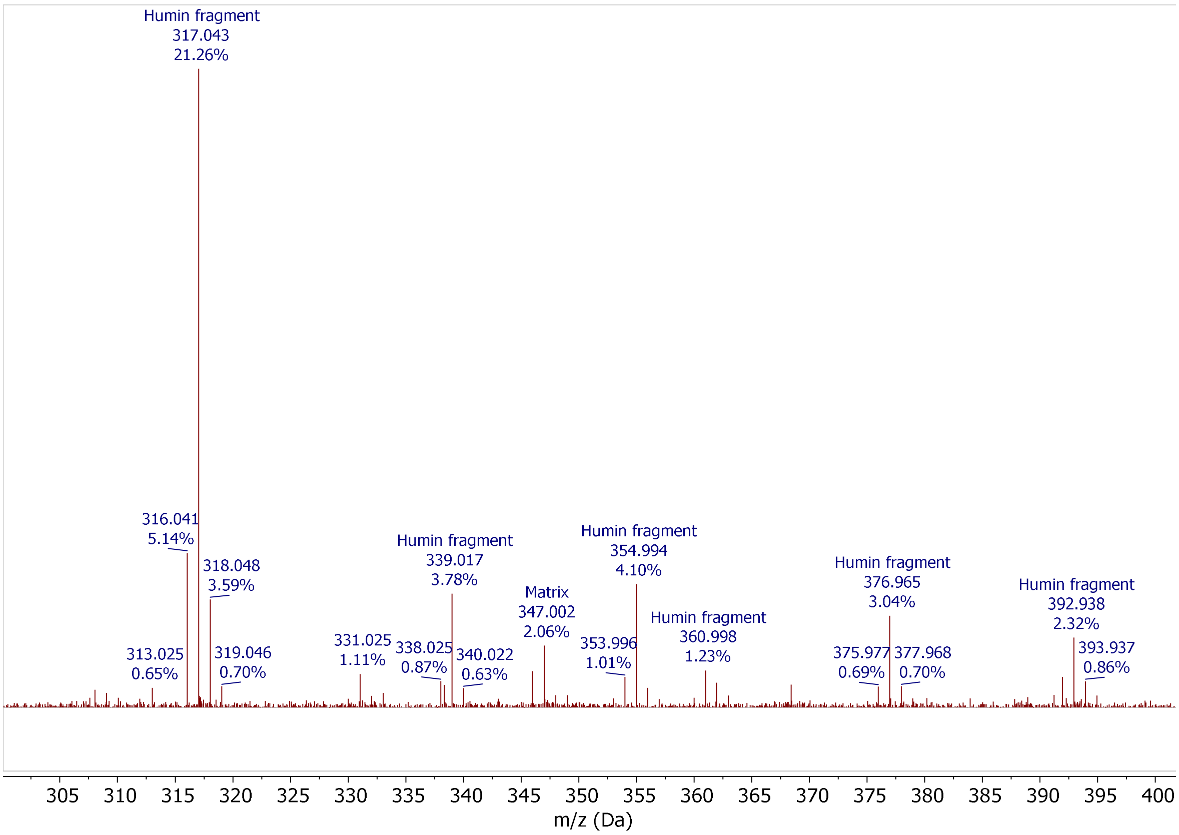

Figure S26: MALDI-TOF-MS spectrum of F3 zoomed in

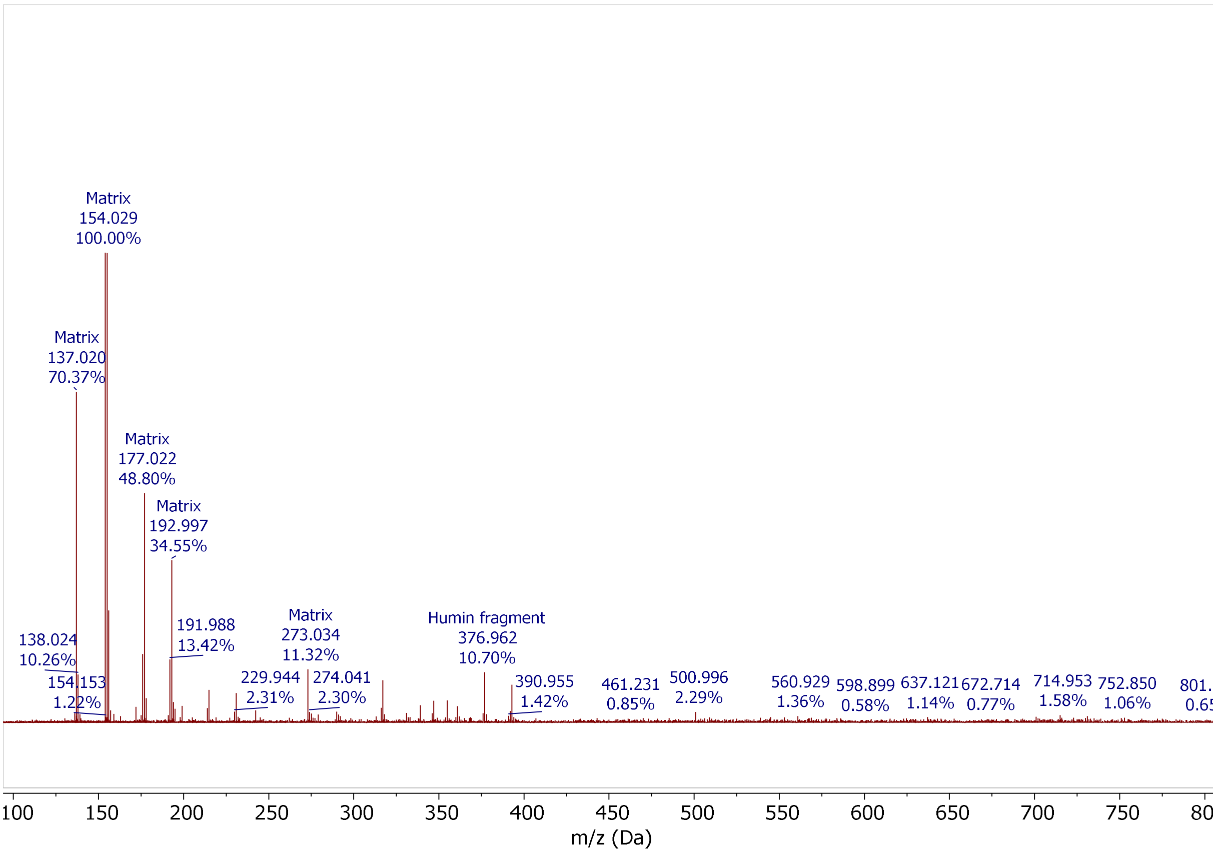

Figure 27: MALDI-TOF-MS spectrum of G1

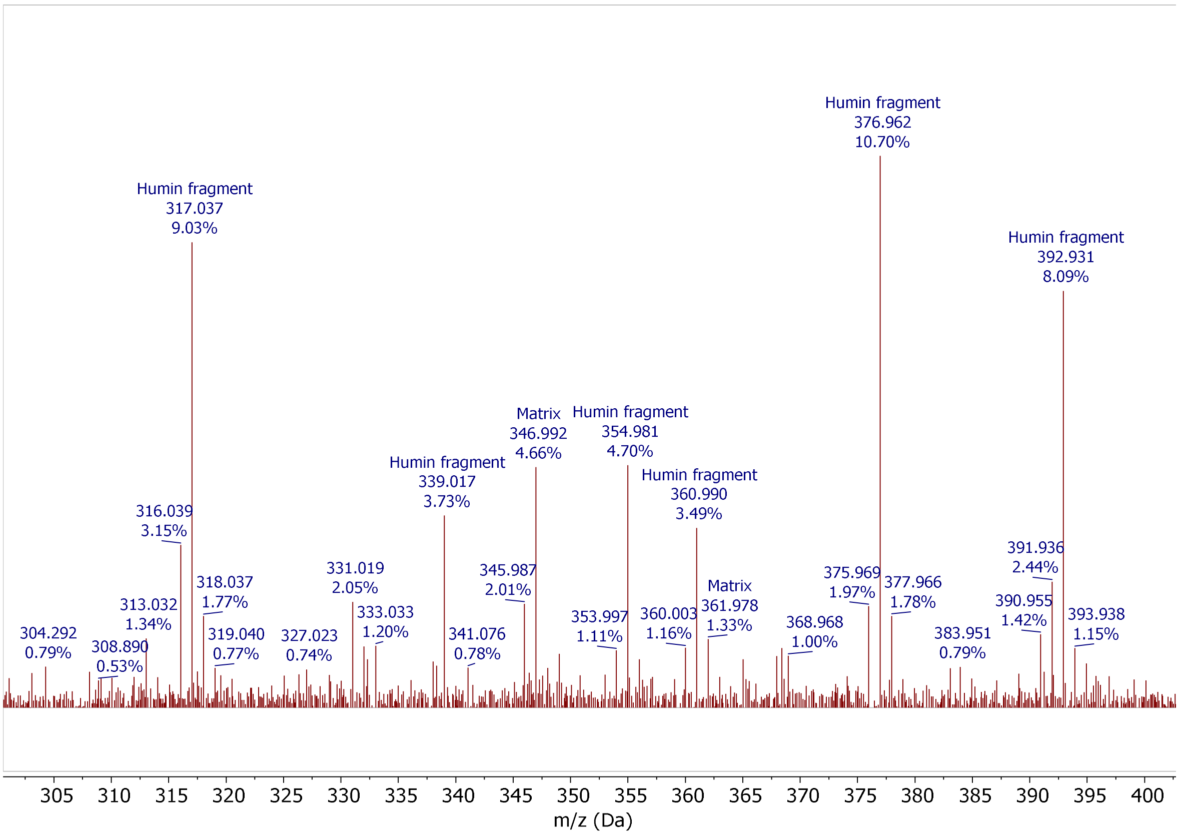

Figure S28: MALDI-TOF-MS spectrum of G1 zoomed in

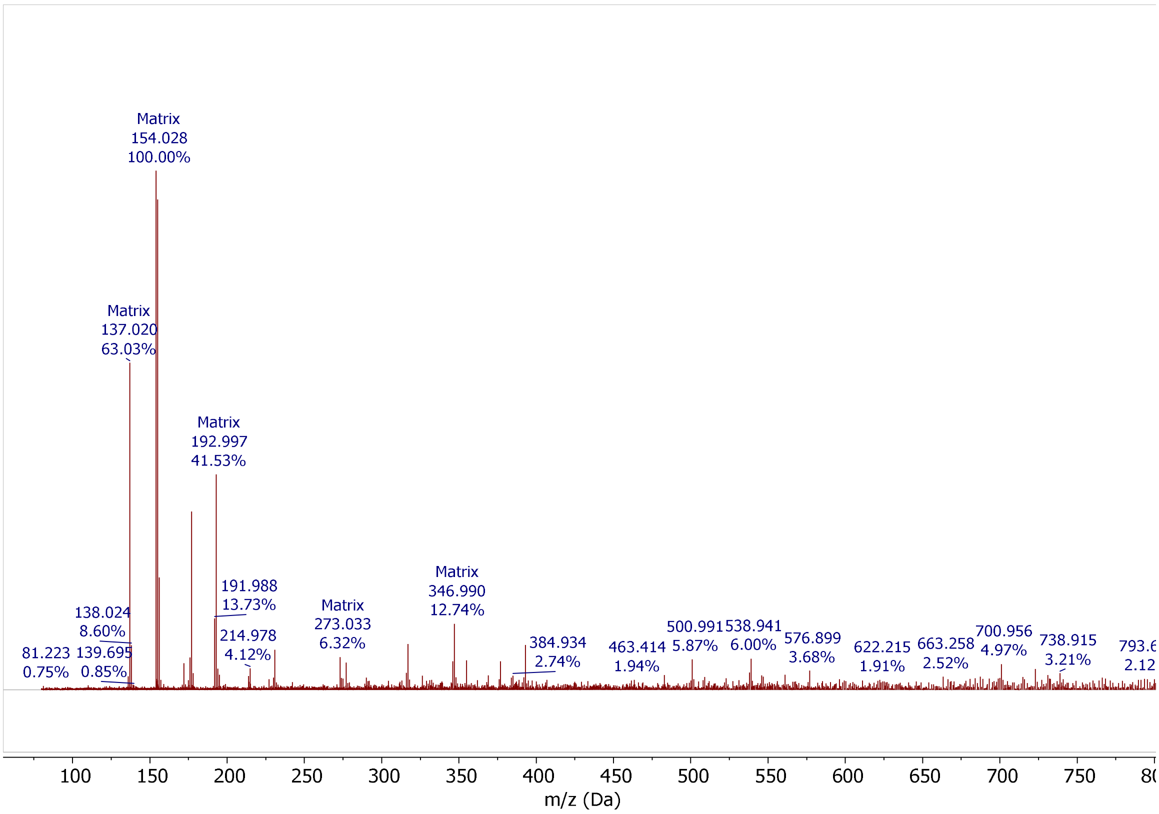

Figure S29: MALDI-TOF-MS spectrum of X1

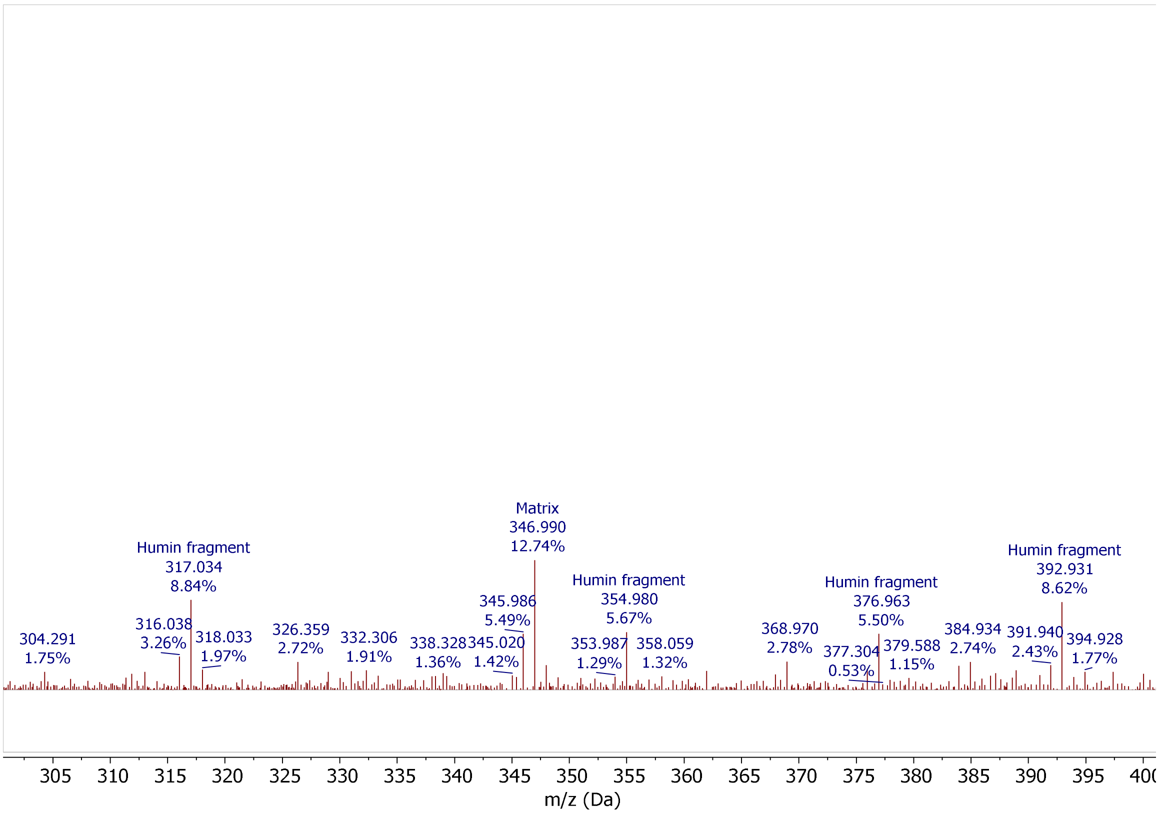

Figure S30: MALDI-TOF-MS spectrum of X1 zoomed in

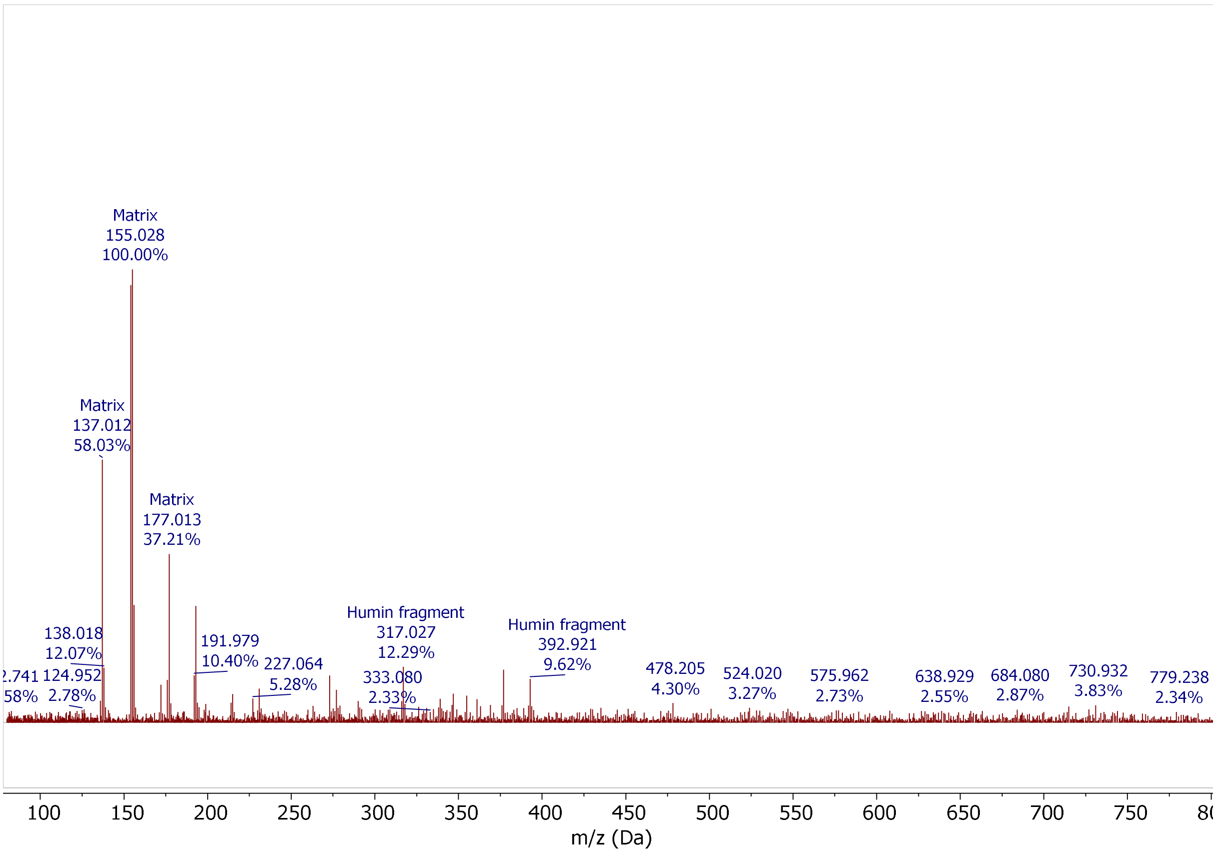

Figure S31: MALDI-TOF-MS spectrum of S1

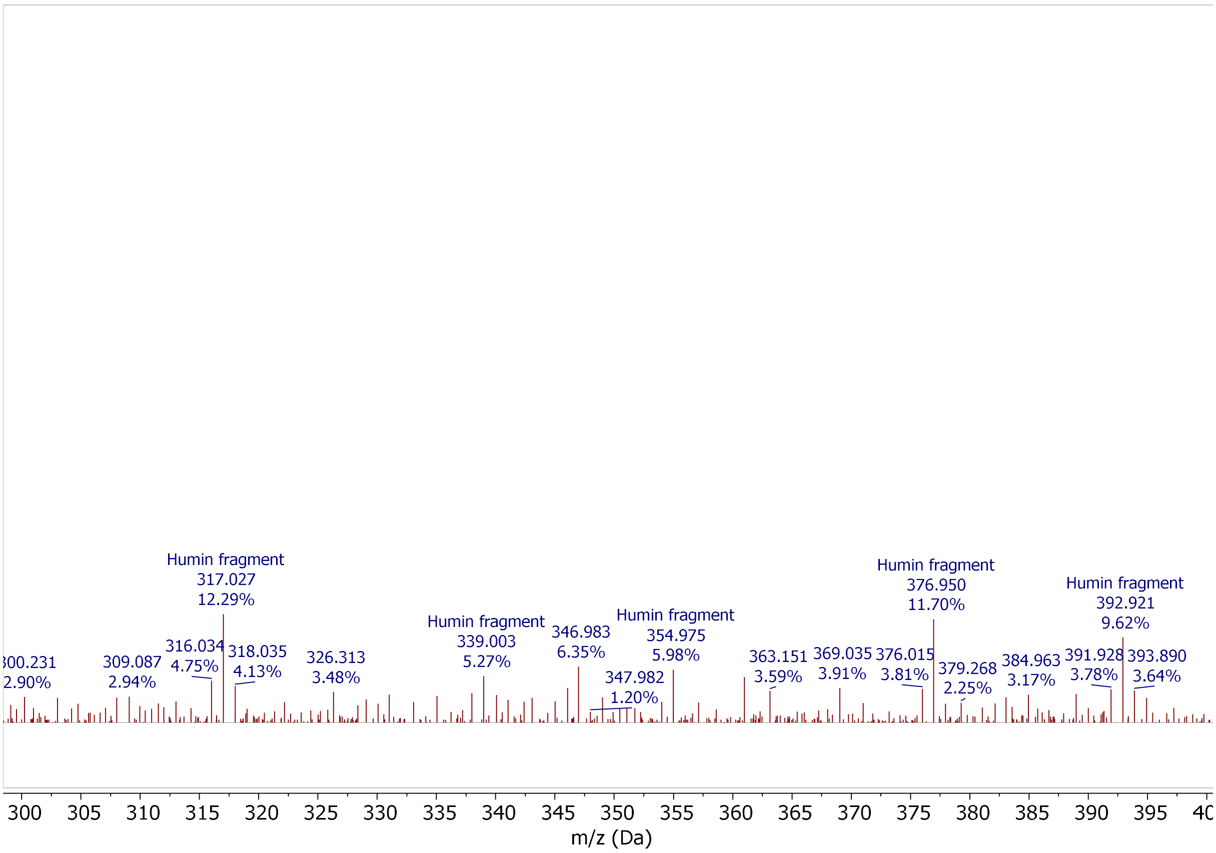

Figure S32: MALDI-TOF-MS spectrum of S1 zoomed in

Electron microscopy

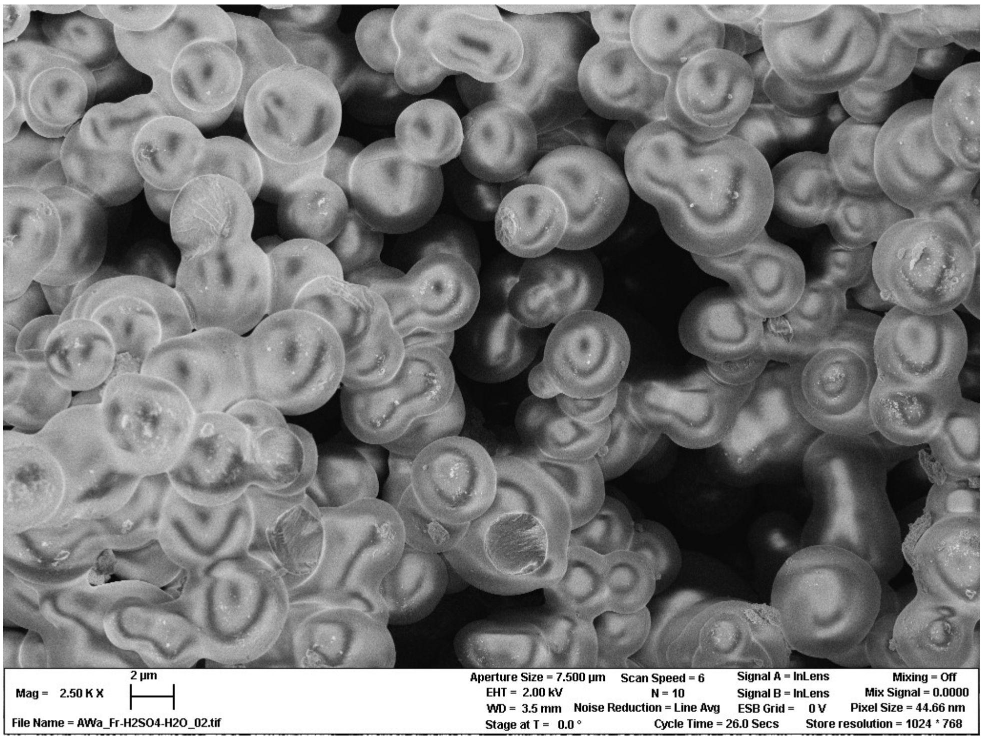

Figure S33: SEM micrograph of F1 zoomed in

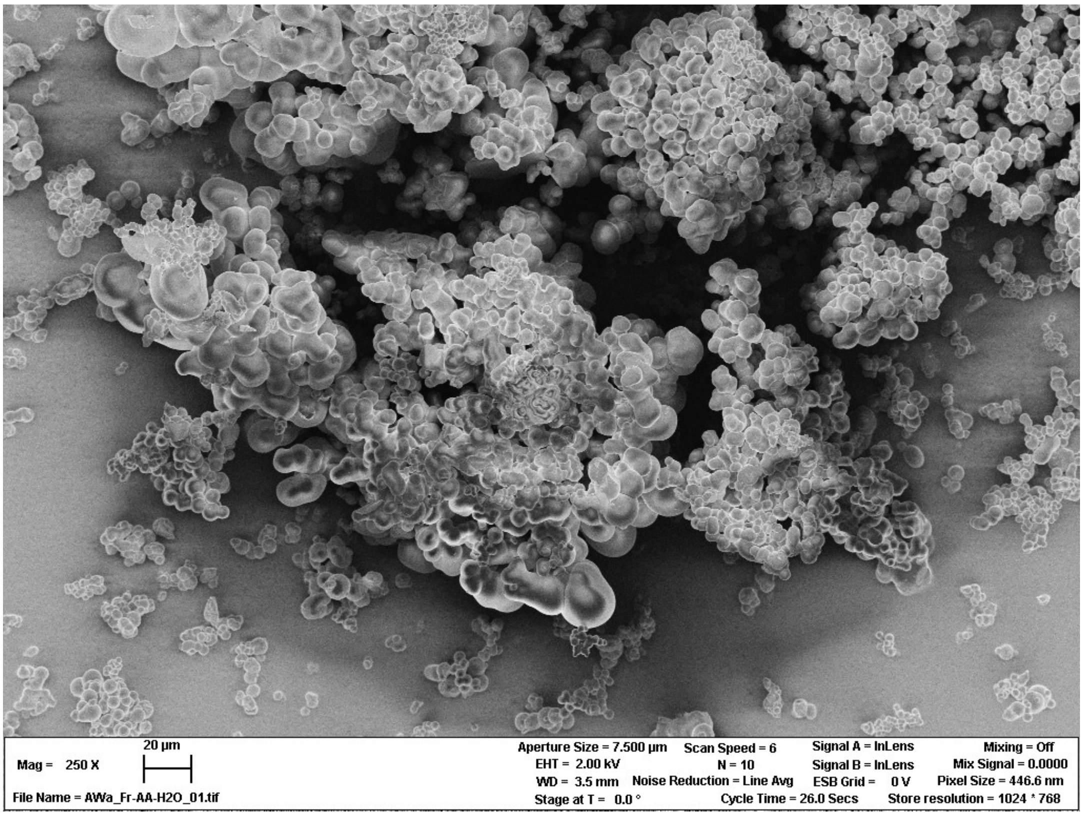

Figure S34: SEM micrograph of F2

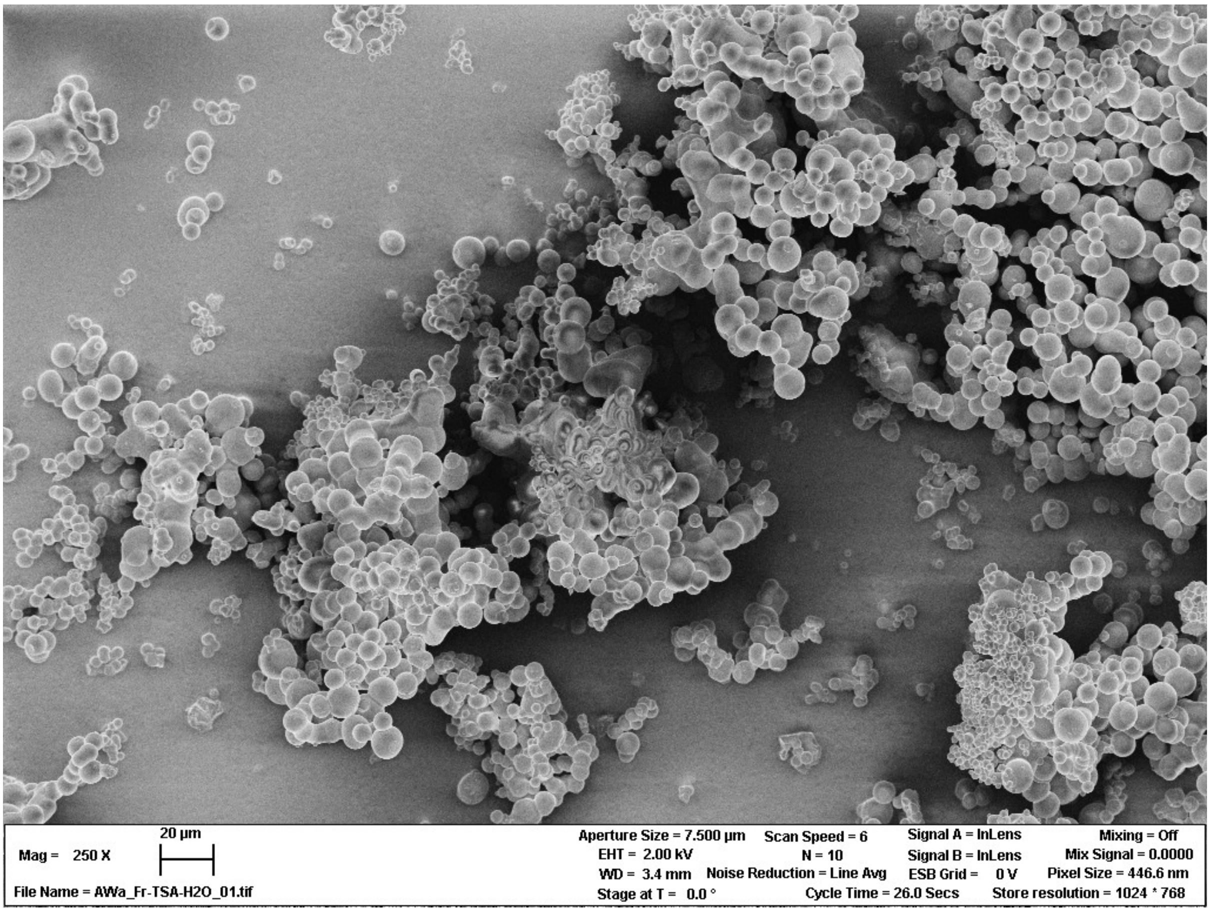

Figure S35: SEM micrograph of F3

Humin conversion

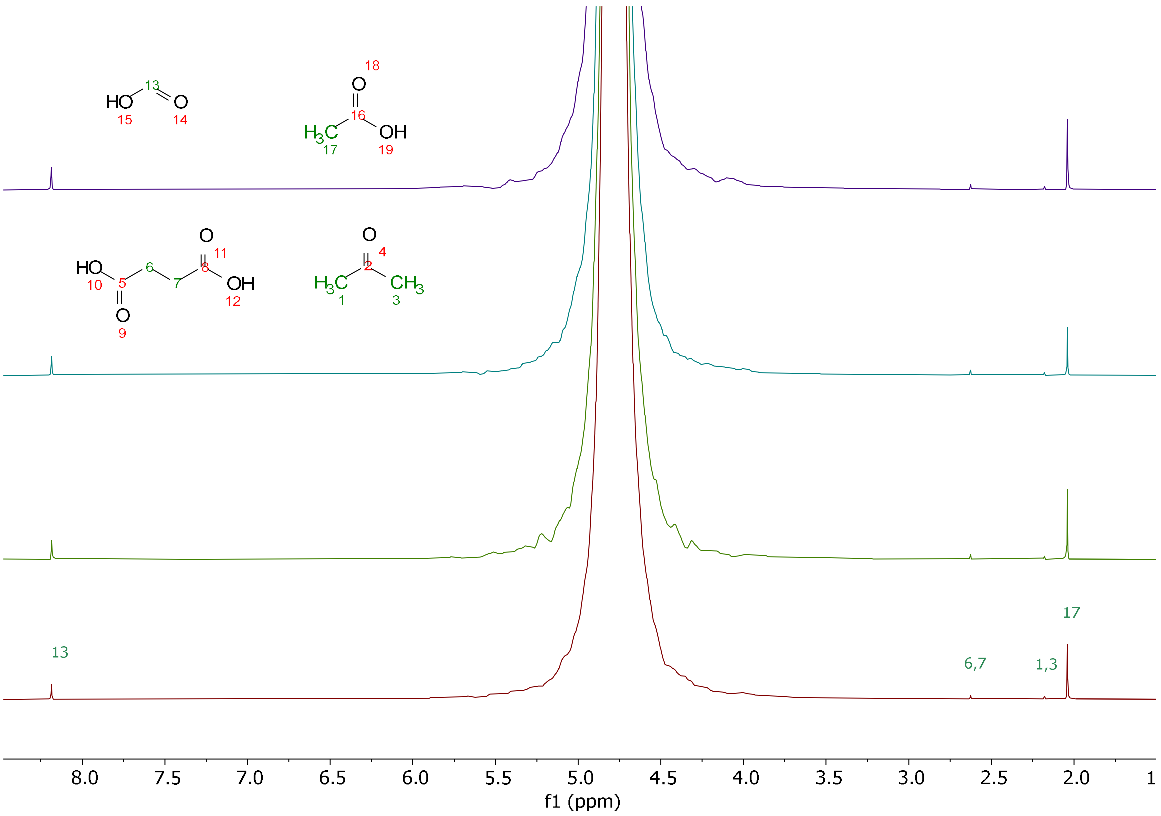

Figure S36: ^{1}H-NMR of the reaction solutions of F1, G1, X1 and S1 after conversion

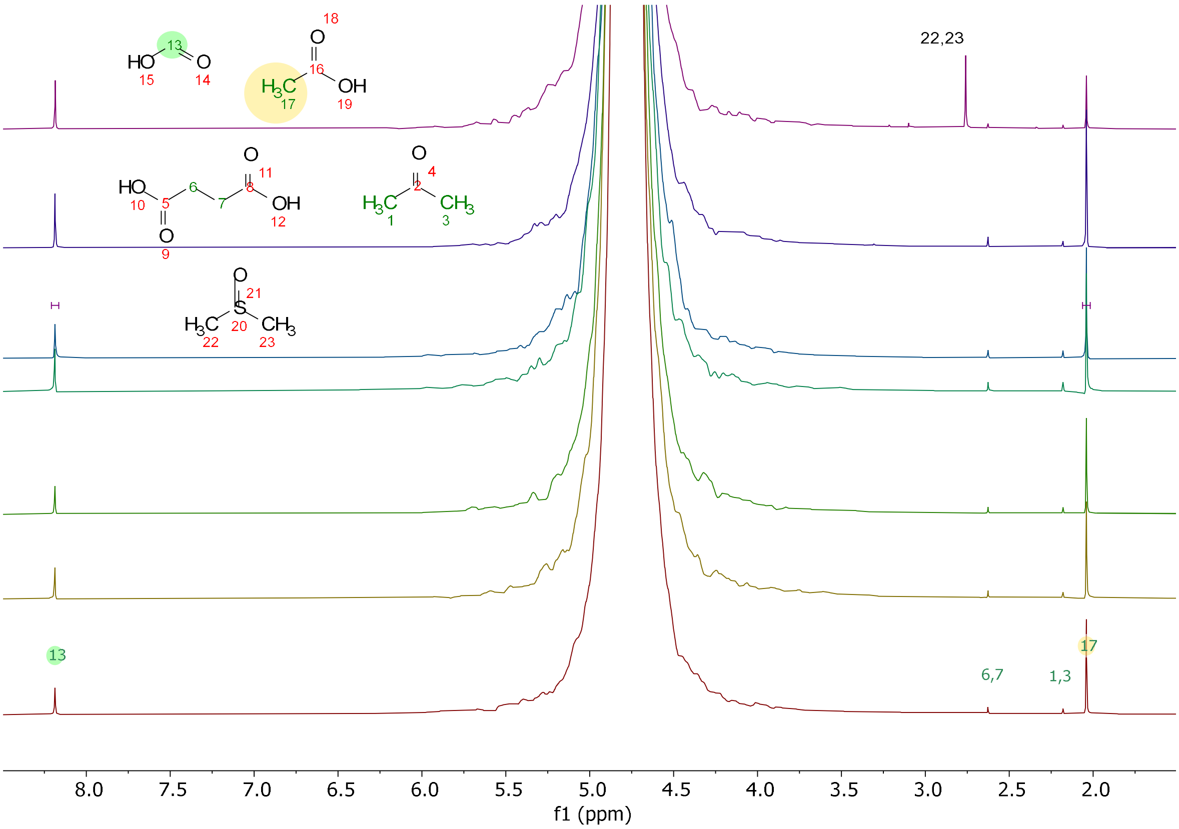

Figure S37: ^{1}H-NMR of the reaction solutions of the fructose humins after conversion

HPLC Analysis

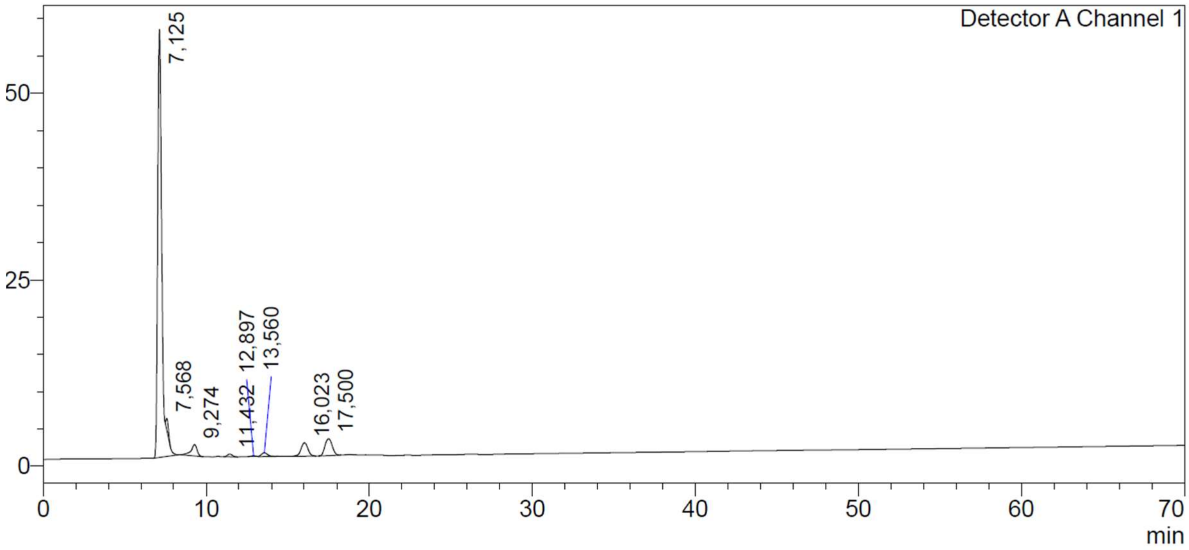

Figure S38: Chromatogram of F1

Table S8: HPLC data of reactions solutions after conversion

	**Concentration [mM]**		
	**Succinic Acid**	**Formic Acid**	**Acetic Acid**
**F1**	1	19	15
**F2**	1	18	14
**F3**	1	20	14
**F4**	1	34	22
**F5**	1	37	24
**F6**	1	30	19
**F7**	1	30	8
**G1**	1	22	18
**X1**	1	21	12
**S1**	1	24	17

Elemental Analysis

Table S9: Product yields and conversion of humins after catalyzed oxidation

	**F1**	**F2**	**F3**	**F4**	**F5**	**F6**	**F7**	**G1**	**X1**	**S1**
**Succinic acid**	0.007	0.008	0.008	0.008	0.007	0.008	0.008	0.008	0.008	0.008
**Formic acid**	0.036	0.040	0.034	0.065	0.055	0.074	0.063	0.043	0.040	0.046
**Acetic acid**	0.056	0.055	0.053	0.091	0.069	0.095	0.033	0.069	0.045	0.065
**DMSO**	0.000	0.000	0.000	0.000	0.000	0.000	0.043	0.000	0.000	0.000
**CO2**	0.388	0.349	0.445	0.382	0.313	0.464	0.158	0.459	0.355	0.469
**CO**	0.000	0.000	0.004	0.012	0.014	0.009	0.003	0.006	0.006	0.009
**Solid residue**	0.512	0.548	0.457	0.442	0.542	0.351	0.691	0.415	0.546	0.395
**Combined Yield**	0.488	0.452	0.543	0.558	0.458	0.649	0.309	0.585	0.454	0.605

Table S10: CHSO of humins synthesized in sulfuric acid and water after catalyzed oxidation

**Elements**	**F1**	**G1**	**X1**	**S1**
**H**	4,17	4,1	4,18	4,135
**C**	57,63	55,59	58,625	56,61
**O**	38,2	38,29	34,245	38,245
**S**	0	0	0	0
**Sum**	100	97,98	97,05	98,99
**H/C**	0,87	0,89	0,86	0,88
**O/C**	0,50	0,52	0,44	0,51

Table S11: CHSO of fructose humins after catalyzed oxidation

**Elements**	**F1**	**F2**	**F3**	**F4**	**F5**	**F6**	**F7**
**H**	4,17	4,02	3,66	4,1	4,835	3,985	3,915
**C**	57,63	55,58	55,735	56,65	62,79	52,175	54,055
**O**	38,2	37,25	38,16	36,285	30,66	39,89	33,665
**S**	0	0	0	0	0	0	5,59
**Sum**	100	96,85	97,555	97,035	98,285	96,05	97,225
**H/C**	0,87	0,87	0,79	0,87	0,92	0,92	0,87
**O/C**	0,50	0,50	0,51	0,48	0,37	0,57	0,47

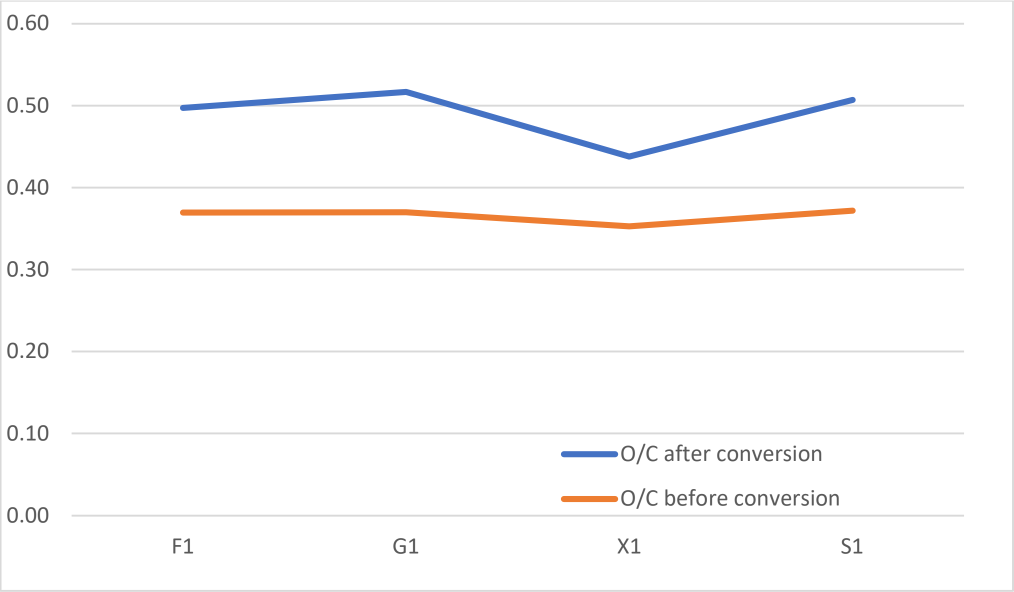

Figure S39: Comparison of O/C ratios before and after catalytic oxidation of humin synthesized in sulfuric acid and water

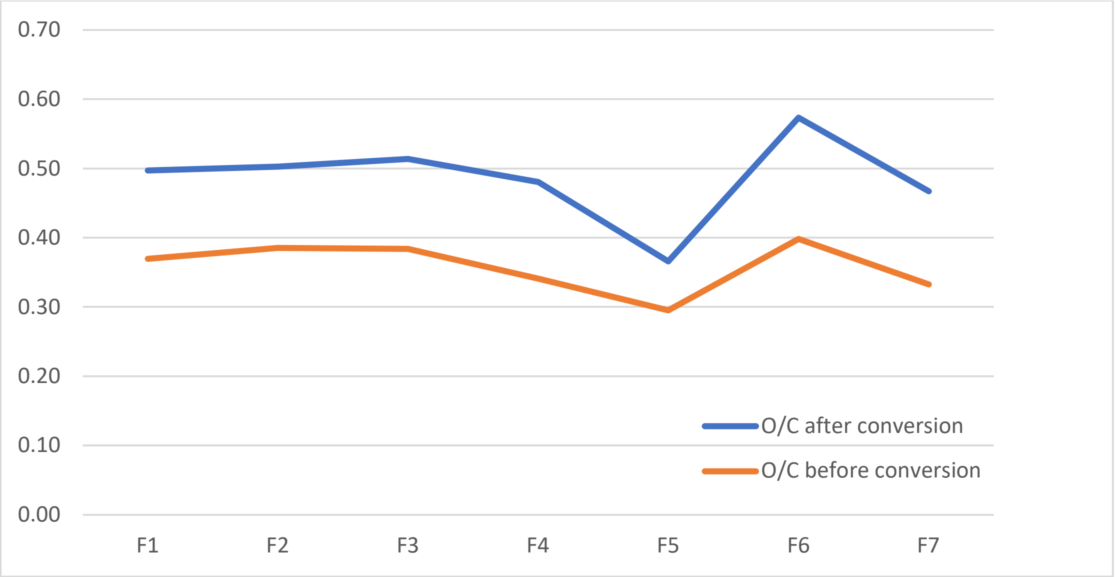

Figure S40: Comparison of O/C ratios before and after catalytic oxidation of fructose humins

IR spectra

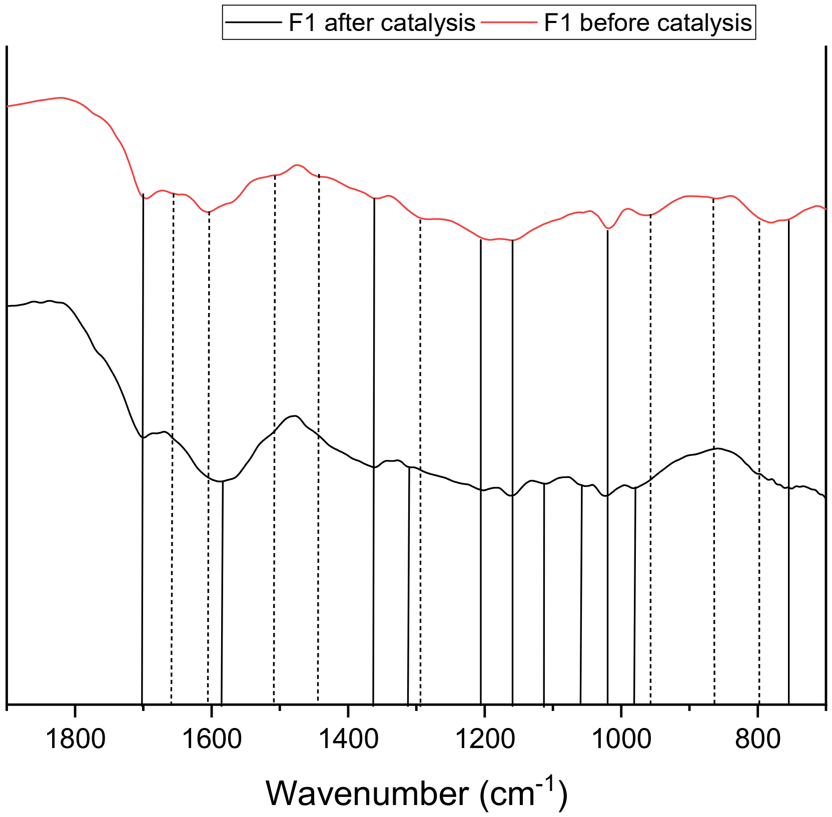

Figure S41: IR-spectrum of F1 before and after the catalysis

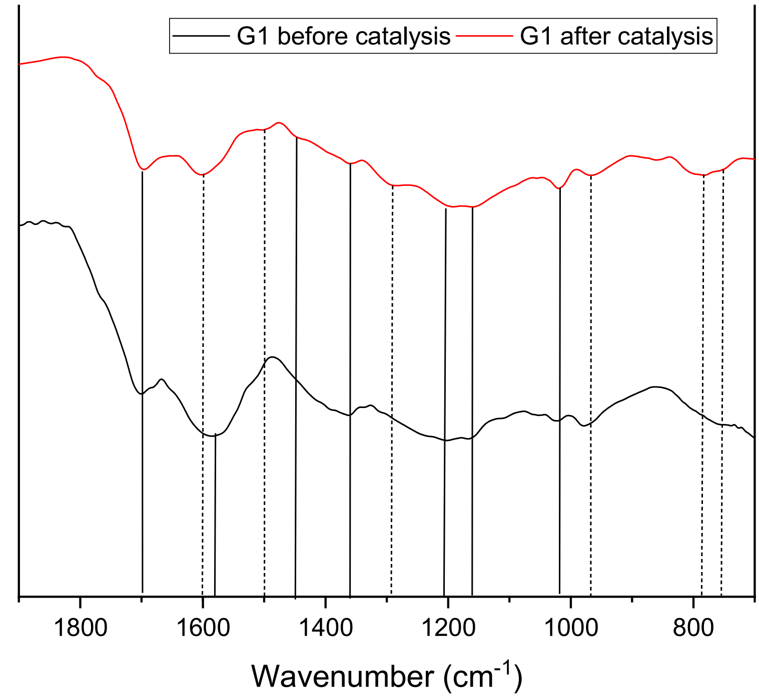

Figure S42: IR-*spectrum of G1 before and after the catalysis*

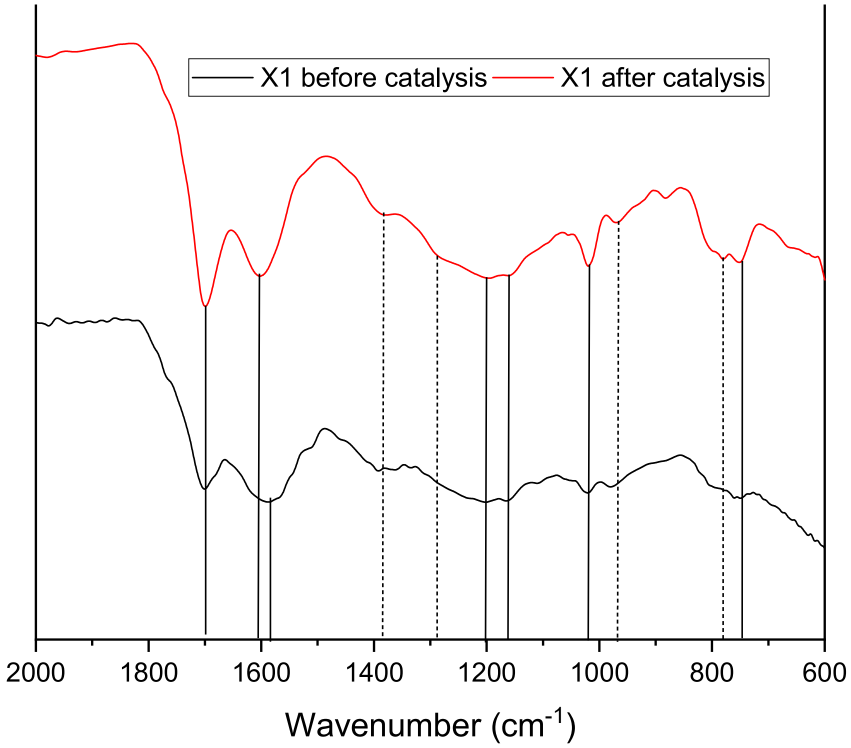

Figure S43: IR-spectrum of X1 before and after the catalysis

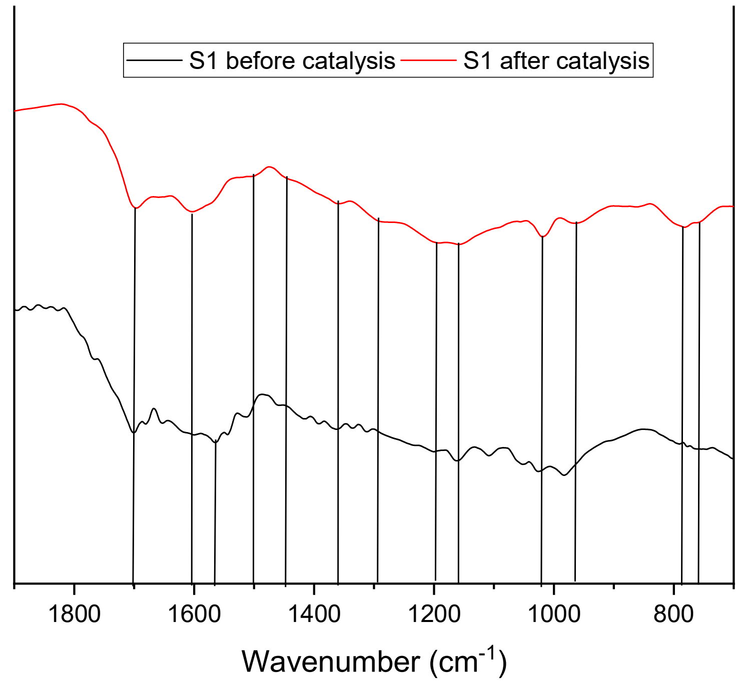

Figure S44: IR-spectrum of S1 before and after the catalysis

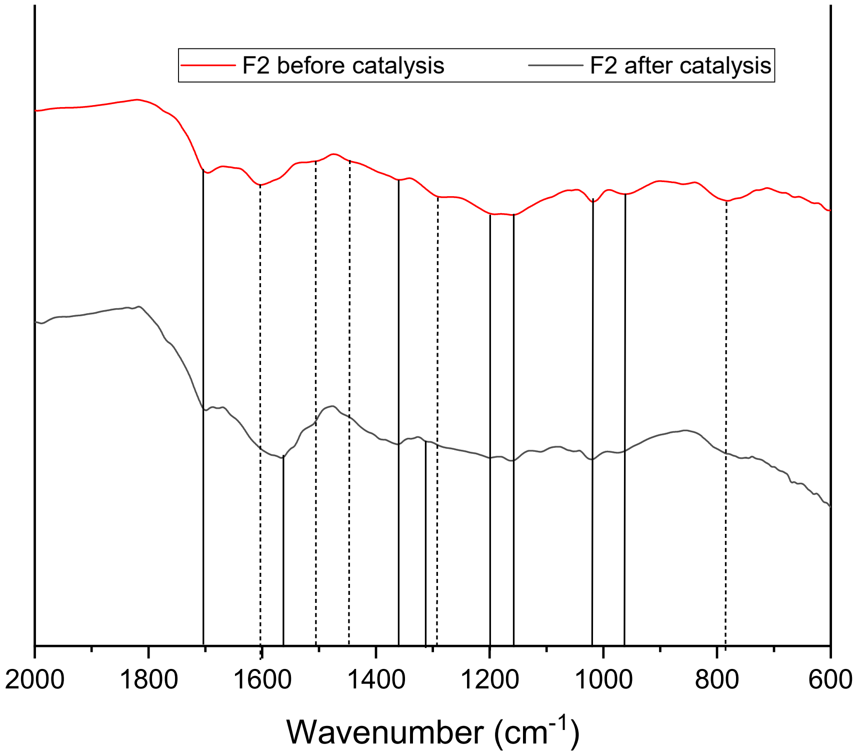

Figure S45: IR-spectrum of F2 before and after the catalysis

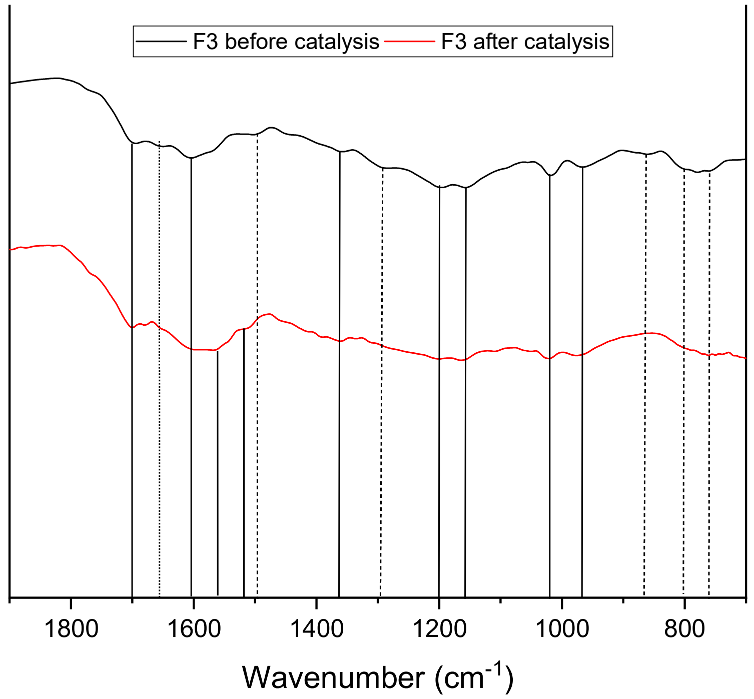

Figure S46: IR-spectrum of F3 before and after the catalysis

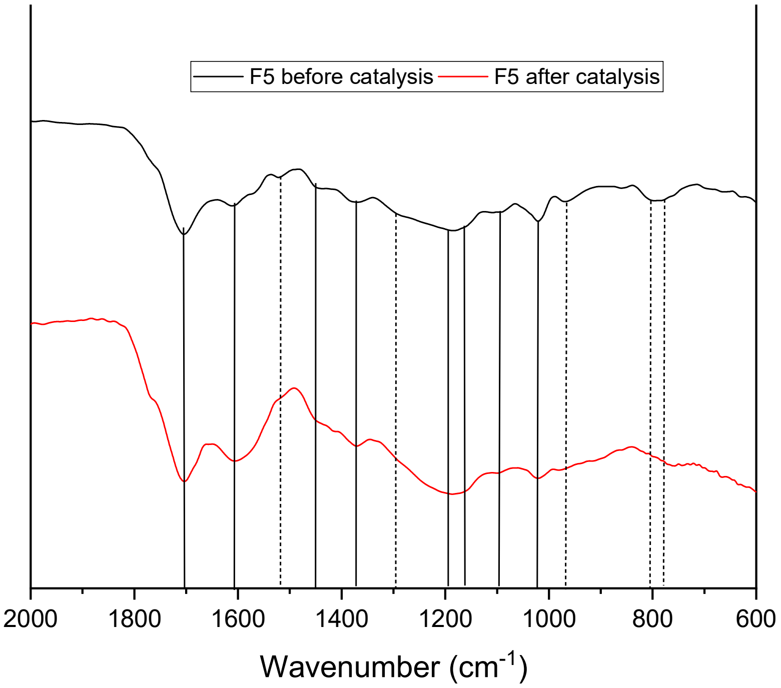

Figure S47: IR-spectrum of F5 before and after the catalysis

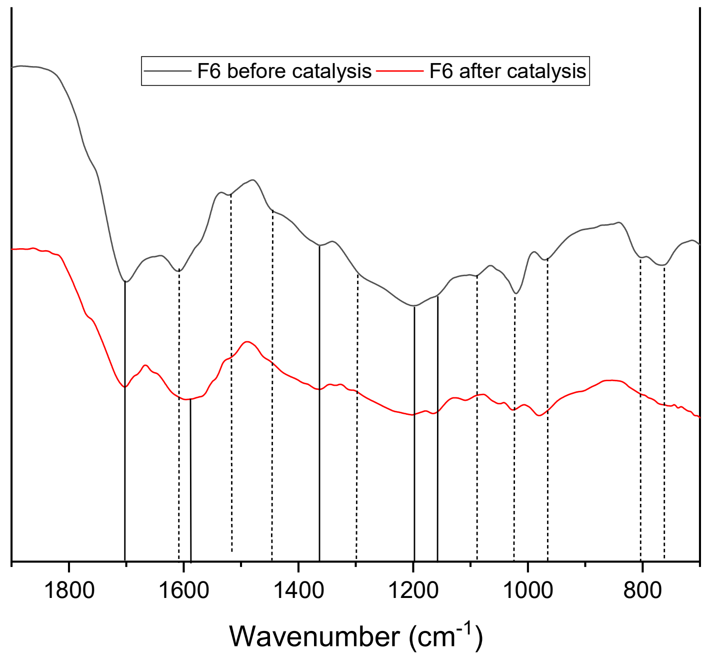

Figure S48: IR-spectrum of F6 before and after the catalysis

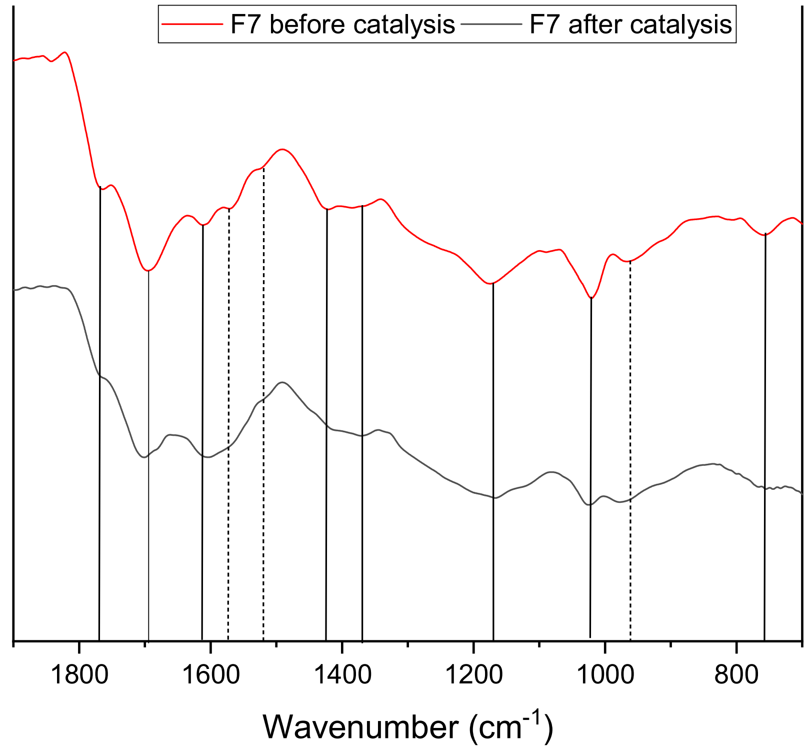

Figure S49: IR-spectrum of F7 before and after the catalysis

Equations used for yield calculations

The conversions of sugars x_i were determined through equation 1:

$$x_i = \frac{c_{i,0} - c_i}{c_{i,0}} * 100 \quad \text{(S1)}$$

With $c_{i,0}$ being the concentration before and c_i the concentration after reaction.

The carbon mass yields of the humin syntheses $Y_{Cmass\%,Hi}$ were calculated using equation 2:

$$Y_{Cmass\%,Hi} = \frac{m_{Hi} * C_{Hi}}{m_{Si} * C_{Si}} * 100 \quad \text{(S2)}$$

With C_{Hi} being the mass percentage of carbon and m_{Hi} the mass of the product humins and m_{Si} and C_{Si} being the respective metrics for the educt sugars.

The yields of the reaction products Pi of humin conversion $Y_{mass,\ Pi}$ were determined with the following equation:

$$Y_{mass,\ Pi} = \frac{m_{Pi}}{m_{Pi,max}} \quad \text{(S3)}$$

With m_{Pi} being the mass of the products and $m_{Pi,max}$ being the maximum possible mass.

m_{Pi} was calculated as the product of the concentration and the molar mass of the reaction product multiplied with the measured density of the reaction solution, to account for deviations in the density through the different densities of the reaction products.

$m_{Pi,max}$ was calculated using equation 4:

$$m_{Pi,max} = \frac{n_{Hi,C}/N_{C,Pi} * M_{Hi}}{m_{Hi} + m_{HPA-5} + m_{H_2O}} \quad \text{(S4)}$$

$N_{C,Pi}$ here is equivalent to the number of carbons contained in the reaction product and $n_{Hi,C}$ is the amount of carbon contained in the humins used for conversion. $n_{Hi,C}$ was determined using equation 5:

$$n_{Hi,C} = \frac{C_{Hi} * m_{Hi}}{M_{Carbon}} \quad \text{(S5)}$$
